# Evaluation of the implementation process of a new emergency triage system: West coast system for triage (WEST)

**DOI:** 10.1371/journal.pone.0350323

**Published:** 2026-06-11

**Authors:** Samah Habbouche, Eric Carlström, Per-Arne Svensson, Carl Magnusson, Lina Dahlen Holmqvist

**Affiliations:** 1 Gothenburg Emergency Medicine Research Group (GEMREG), Gothenburg, ‌‌Sweden; 2 Department of Internal Medicine and Clinical Nutrition, Institute of Medicine, Sahlgrenska Academy, University of Gothenburg, Gothenburg, Sweden; 3 Institute of Health and Care Science, Sahlgrenska Academy, University of Gothenburg, ‌‌Gothenburg, Sweden; 4 Department of Molecular and Clinical Medicine, Institute of Medicine, Sahlgrenska Academy, University of Gothenburg, Gothenburg, Sweden; Stamford Health System: Stamford Hospital, UNITED STATES OF AMERICA

## Abstract

**Background:**

Emergency Department (ED) crowding has escalated globally and has been associated with delayed treatment and increased short-term mortality. In western Sweden, several EDs transitioned from the Rapid Emergency Triage and Treatment System (RETTS©) to the West coast system for triage (WEST), based on principles of the South African Triage Scale. While WEST was introduced and spread rapidly and may have potential benefits, reports suggested challenges during its implementation.

**Objective:**

This study examined the implementation of WEST at the first two EDs (ED1 and ED2) and identified barriers and facilitators. The study focused on the transition from RETTS© to WEST and explored staff experiences across implementation stages.

**Methods:**

The study used a qualitative descriptive design with a mixed-methods approach and included semi-structured individual interviews with 36 triage nurses from two hospitals (ED1 n = 17; ED2 n = 19). Interviews explored experiences with WEST, implementation challenges, and comparisons with RETTS©, using Rogers’ “Diffusion of Innovations” as a sensitizing framework and treating each ED as a distinct social system. A deductive qualitative content analysis guided by Rogers’ Diffusion of Innovations was conducted, with inductive coding of unexpected themes from the final open question. Descriptive analyses of Likert-scale items complemented the qualitative analysis. The study received ethical approval, and written informed consent was obtained from all participants before interviews.

**Results:**

ED1 participants expressed great enthusiasm and perceived WEST as a notable improvement. ED2 responses were more varied, with reservations commonly targeting the implementation process and concurrent organizational changes. Across both sites, most nurses (30/36) perceived WEST as more accurate than RETTS© in identifying critically ill patients.

**Conclusions:**

Implementing WEST was feasible but context dependent. Information, motivation, and consensus-building, education, and organizational readiness emerged as important factors in the implementation process. Viewing each ED as a distinct social system, with its own culture and readiness for change, helped explain why adoption of WEST varied between sites.

## Background

Emergency Department (ED) crowding is a persistent global public health challenge [1]. Rising attendances and suboptimal use of hospital resources mean that demand often exceeds capacity, with crowding linked to delays in life-saving treatments, increased complications, and higher 30-day mortality [2–5]. In Sweden, where the data for this study was collected, waiting times have increased over the past three decades [1]. Improvement efforts have therefore targeted ED flow, including patient prioritization systems [6,7]. This study examined the early implementation of a new triage system at two EDs. The new triage system was designed to alleviate a severe ED crowding situation. By capturing the experiences of triage nurses during the transition from the old triage system to the new one, the study identified organizational facilitators and barriers that can inform future system changes in similar contexts.

Rapid Emergency Triage and Treatment System (RETTS©) is a widely used triage system in Sweden. The methodology and structure of RETTS© is described in detail elsewhere [8,9]. However, concerns have been raised regarding its reliability, particularly in the assignment of acuity levels [10]. Acuity refers to the severity or urgency of a patient’s condition and determines how quickly they need medical attention. Sometimes over-triage occurs, defined as assignment of a higher acuity level than clinically warranted, which increases monitoring burdens and risks diverting attention from truly high-risk patients [11]. In an educational evaluation, inter-rater agreement among RETTS© users was moderate, particularly in assessments requiring clinical judgment, indicating gaps in training and consistency [12]. In response to perceived over-triage, a hospital in western Sweden reviewed its triage procedures and developed West coast system for triage (WEST), drawing on principles from the South African Triage Scale [13,14]. WEST, outlined in detail in earlier work [14], considered four domains: (i) vital parameters summarized via National Early Warning Score 2 (NEWS2), (ii) predefined warning signs and symptoms, (iii) additional care needs (e.g., frailty), and (iv) structured clinical judgment [14,15]. Unlike RETTS©, where a single abnormal parameter could escalate acuity level, WEST emphasized patterns of abnormality and integrated clinical judgment. Prior work on WEST yielded the anticipated reduction in over-triage [14]. While the rationale for developing WEST was grounded in concerns about over-triage and consistency in RETTS, introducing a new triage system requires substantial changes to workflows, roles, and shared clinical judgment. Accordingly, the success of WEST depends not only on its design but also on how it is implemented and adopted in everyday ED practice.

Organizational changes in healthcare often fail due to limited planning, weak motivation, and change fatigue [16,17]. Subcultures can resist new systems, underscoring the importance of deliberate strategies for change [18]. However, few studies provide in-depth assessment of health professionals’ reactions to implementation in an ED context and to the best of our knowledge no previous studies have investigated health professionals’ responses to introducing a new triage system in Sweden [17,19]. One study from a Danish ED found that implementation was non-linear and context-dependent, with staff primarily focused on ensuring the best possible acute patient pathway for their own patients [17]. Process improvement approaches in ED operations identified three main barriers 1) difficulties to generalize results from other areas, 2) medical staff resistance to change and 3) increase in costs [20]. These findings collectively underscore the complexity of implementation in ED settings and resonate with the present study’s focus on how local context and staff perceptions shape adoption outcomes.

### Theoretical framework

**Fig 1:**
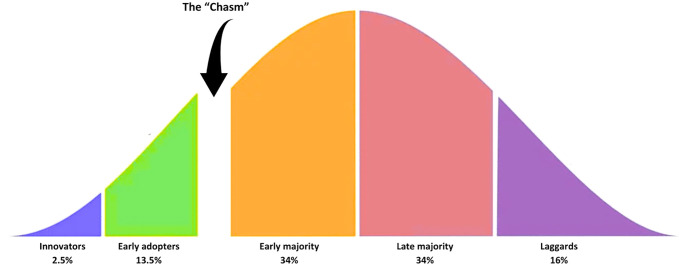
Conceptual illustration adapted from Moore’s Crossing the Chasm (1991). Adoption curve adapted from Rogers’ “Diffusion of Innovations” and Moore’s concept of “crossing the chasm,” illustrating the challenge of moving from early adopters to the early majority. Bridging this gap is essential for innovations to achieve mainstream adoption.

In this study, “Diffusion of Innovations” based on Rogers served as a sensitizing framework to structure inquiry and interpretation. “Diffusion of Innovations” describes how innovations spread over time through communication channels within a social system, influenced by innovation attributes and adopter categories [21]. Rogers describes five adopter categories along a bell-shaped curve: innovators, early adopters, early majority, late majority, and laggards, each reflecting different degrees of innovativeness and willingness to embrace change [21]. Rogers’ concepts of early and late adopters have been applied across a range of healthcare change contexts, from studying how financial rewards influence whether hospitals adopt new practices early or late, to challenge and refine the traditional adopter categories in the context of health technology and to assess whether clinical teams are organizationally ready to implement new care programs [22–24]. Rogers’ concepts are particularly useful in EDs because EDs function as unique social systems and operate within their own specific contextual factors, including staff dynamics, leadership styles, patient demographics, and resource availability. As distinct social systems, EDs experience rapid changes in protocols and practices based on evolving patient needs, staffing, and technology [25]. Furthermore, Rogers’ “Diffusion of Innovations” offers concepts for understanding adoption within a social system [21,26]. As with many implementation efforts in emergency care, progressing from initial uptake to broad and sustained routine use can be challenging, particularly during the transition from early adopters to the early majority, which Moore describes as “Crossing the Chasm” (Fig 1) [27]. “Crossing the Chasm” highlights the crucial divide between these two essential groups in the implementation life cycle: the early adopters and the early majority. Those in the early adopter category are often visionaries who are eager to embrace new technologies, due to their recognition of potential benefits. In contrast, the early majority tend to adopt a more cautious approach, seeking evidence of reliability before making a commitment [21,27,28]. Together, these frameworks shaped the study design and guided data collection and interpretation, with a particular focus on nurses’ perceptions, organizational readiness, and early adoption dynamics during WEST implementation.

## Aim

The study had two aims: (i) to explore how triage nurses experienced the transition from RETTS© to WEST, and (ii) to describe perceived benefits and drawbacks of WEST relative to RETTS© regarding flexibility, clarity, and perceived accuracy. A secondary aim was to compare the implementation processes between ED1 and ED2.

## Materials and methods

### Study design and methodology

This study employed a qualitative descriptive design with a mixed‑methods approach, integrating semi-structured interviews with Likert‑scale summation to capture both narrative insights and structured assessments [29,30]. The interview guide drew on a literature review, “Diffusion of Innovations” theory and previous implementation research. It included items targeting key components of the implementation process, information, motivation and consensus, education, and organizational factors following Janssen et al. [31]. Additional components assessed perceived performance of triage systems, focusing on flexibility, clarity, perceived accuracy and innovation attributes like relative advantage, compatibility, and complexity [21]. Face validity was assessed by two individuals who met the same inclusion criteria as the study participants, ensuring that the interview guide was understandable and relevant for the target group. Interviews strictly followed the guide; clarifications were provided when needed. The final item invited open reflections, allowing unexpected themes to emerge and be coded separately before thematic grouping. The final interview guide is provided as a supplement (S1 File).

### Study context

Sahlgrenska University Hospital, one of Europe’s largest hospitals and the largest in Sweden, employs approximately 17,000 staff and has about 2,300 beds across three hospital sites [32]. It provides emergency and basic care for about 700,000 inhabitants in the Gothenburg region, as well as highly specialized care for approximately 1.7 million residents in western Sweden. ED1 was situated on the tertiary-care campus, whereas ED2 was located on the level 1 trauma center campus. Both EDs managed approximately 55,000 attendances annually. Due to a prolonged problem with ED crowding at the EDs included in this study, a Health Technology Assessment (HTA) of triage systems was initiated. The HTA aimed to identify triage systems effective in supporting patient prioritization and ED flow under crowded conditions [13]. In the present study, RETTS© was compared with WEST. Both systems used five color-coded levels (red, orange, yellow, green, blue).

Based on the HTA findings, WEST was selected as a promising system to support patient flow at ED1 and ED2 under crowded conditions by combining a broader assessment framework with explicit room for clinical judgment. In addition, WEST incorporates structured neurological assessment tools, including the National Institutes of Health Stroke Scale (NIHSS) and the Glasgow Coma Scale (GCS). WEST was implemented at ED1 from September to November 2019. ED2 implemented WEST in September 2020, between the first and second waves of COVID-19. The educational intervention was designed to be identical at both sites and was delivered in the same manner at both EDs, as confirmed by department managers. At the time of the study, WEST was available in a pocket-sized reference card, allowing triage nurses to keep the material readily accessible during triage. Baseline performance differed somewhat between the two sites: ED2 had longer length-of-stay and time-to-physician than ED1, as recorded in the patient administrative systems.

### Study population

Triage nurses from both EDs were interviewed during a two‑week period between April 16 and April 30, 2021. At the time of the interviews, WEST had been implemented for approximately six months at ED2 and for seventeen months at ED1. Inclusion criteria were ≥1 year of triage experience and ≥3 months of WEST use. All registered nurses working in triage at ED1 (N = 34) and ED2 (N = 44) who met the inclusion criteria were invited to participate through consecutive sampling, using e-mail invitations and announcements at workplace meetings. Interested nurses contacted the interviewer directly by email or in person, and written informed consent was obtained prior to each interview (S2 File).

### Data collection

All interviews were conducted face-to-face during work hours in quiet rooms (30–45 minutes) by the first author. A structured field‑note template aligned with the interview guide was used to document responses in real-time in a consistent and systematic manner. Responses were documented in writing in a near-verbatim style as participants spoke; when needed, participants were asked to pause briefly to ensure accuracy. Tone, pauses, and manner of speaking were not recorded. Following each interview, the notes were returned to participants for confirmation through member checking within two weeks, ensuring that their answers had been accurately captured. Once confirmed, the notes were compiled into a dataset for analysis, from which illustrative quotes reflecting the diversity of participants’ perspectives were selected to support interpretation [33,34].

Data saturation was monitored throughout data collection. As interviews progressed, differences between participants’ responses decreased while similarities increased with recurring themes related to information dissemination, training quality, and organizational adjustments converging consistently after approximately 20–25 interviews. Data collection continued beyond this point to ensure all willing participants could contribute and to minimize the risk of overlooking subgroup perspectives, resulting in 36 interviews in total. Reporting follows the Consolidated Criteria for Reporting Qualitative Research (COREQ) (S3 File) [35].

### Reflexivity and interviewer positionality

All interviews were conducted by the first author, a resident physician in emergency medicine at ED2 at the time of data collection, with clinical experience but no formal leadership responsibilities at either site. Several strategies were employed to reduce potential influence on participant responses. Recruitment was self-selected, with nurses approaching the researcher voluntarily, which minimized the risk of participation being shaped by hierarchical pressure or role expectations. A standardized interview guide was followed strictly across both sites, with probing limited to necessary clarifications, and a structured note-taking format was applied to minimize selective capture of responses. Throughout the study, reflexive attention to the interviewer’s positionality was maintained, and potential role-related influences were considered during analysis. To further balance interpretive bias, investigator triangulation and collaborative coding were employed, and negative cases were considered during theme development.

### Data analysis

Demographic data were analyzed descriptively. Likert-scale responses were summarized using proportions of high ratings, defined as scores of 4–5 on a five-point scale. Missing responses were rare and occurred only sporadically, most notably for items related to organizational change; proportions were therefore calculated based on the number of valid responses for each item. No inferential statistical testing was planned or performed given the study design and sample size. A deductive qualitative content analysis was conducted using “Diffusion of Innovations” as a sensitizing framework [36]. Initially, data were coded within each ED, then compared across sites. Two researchers independently coded an initial subset, reconciled discrepancies to refine a codebook, and subsequently recoded the remaining interview notes. Credibility was strengthened through this independent coding process, followed by dialogue to reconcile discrepancies and establish a final coding scheme [37]. No quantitative inter-rater reliability measure was calculated as the analysis aimed to capture the participants’ perspectives and nuances rather than classify discrete units. As interviews were not audio-recorded, analysis was based on near-verbatim field notes as described above.

### Ethical considerations

Participation was voluntary and written informed consent was obtained prior to each interview (S2 File). Only the interviewer had access to raw data, ensuring participant confidentiality throughout the study. The study followed the Declaration of Helsinki and was approved by the Regional Ethical Review Board in Gothenburg (approval number for the initial pilot study on WEST: Dnr1078-18; approval number for the amendment covering the present study: Dnr2020–06571).

## Results

### Study population

In total, 36 of 78 eligible nurses participated (response rate 46%; ED1: 17/34, ED2: 19/44). Participants from ED2 were older (median 43, interquartile range (IQR 31–51) vs median 33, IQR 27–38) and reported longer professional experience (median 10 years, IQR 6–23 vs median 5 years, IQR 3–8). Gender distribution (female/male) was similar between ED1 (12/5) and ED2 (14/5) (Table 1).

**Table 1:** Characteristics of the study population.

	ED1	ED2
Participants, n/N (%)	17/34 (50%)	19/44 (43%)
Age, median (IQR)	33(27-38)	43(31-51)
Years in profession, median (IQR)	5(3-8)	10(6-23)
Female/male	12/5	14/5

*IQR= Interquartile range

### Overall response

Descriptive summaries of Likert-scale responses showed that ED1 respondents generally reported positive perceptions of WEST as a working tool, reflected by high agreement or strong agreement across ratings. Responses from ED2 tended to be more variable across participants; however, when evaluating perceived accuracy most participants across both sites agreed or strongly agreed that WEST was more accurate than RETTS© in identifying severely ill patients.

### The change (implementation of WEST)

**Fig 2:**
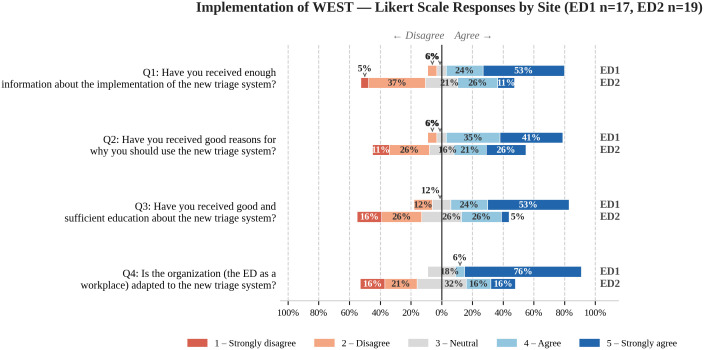
Responses related to the implementation of WEST. Distribution of Likert-scale responses (1–5) to questions related to the implementation of WEST at ED1 and ED2. Percentages may not sum to 100% due to rounding.

Differences in the process of implementing WEST were observed across all implementation domains at the two EDs, including information, motivation, education, and organization (Fig 2). ED1 respondents tended to report higher Likert-scale ratings than ED2 respondents.

### Information strategies

A majority of participants at ED1 (76%) agreed or strongly agreed that the information provided about the new triage system was sufficient, compared with 37% at ED2 (Fig 2).

*“We were informed months before the implementation on many different occasions, via e-mail and verbally.”* (Nurse, ED1)

Criticism from ED2 focused on the perception that the implementation team did not adequately communicate information. Rumors circulated that ED1 had already introduced WEST, and that ED2 would be next.

*“There was a situation of non-existent information that nurtured rumors at the ED.”* (Nurse, ED2)

ED2 participants expressed a need for clear information and requested briefings and platforms to voice concerns. Participants also reported that information was lacking for physicians within the ED and in connected units.

### Motivational strategies

In ED1, 76% of participants agreed or strongly agreed that they were motivated by perceived limitations of RETTS©, particularly its tendency to over-prioritize patients, compared with 47% in ED2.

*“We have a lot of over-prioritizations, and the WEST system reduces the number of patients prioritized at the orange triage level. It would facilitate the identification of the most severely ill individuals.”* (Nurse, ED2)

Nurses at ED2 reported that the implementation was perceived as poorly received by staff, which they felt resulted in low motivation to adopt the new system. They described insufficient background information regarding the rationale for change. The simultaneous introduction of new laboratory routines, also perceived as poorly communicated, further reduced motivation and left staff feeling unprepared.

*“The reduction in laboratory services, driven by cost-saving measures, along with the introduction of new routines without consulting the ED staff, is not very motivating.”* (Nurse, ED2)

### Educational strategies

Participants’ perceptions of education differed substantially between sites. At ED1, 76% of nurses agreed or strongly agreed that they had sufficient time for training and appreciated opportunities to provide feedback, compared with 32% at ED2.

*“The WEST education included seminars and discussion. We got the opportunity to get help in the triaging process with a supervisor during the first weeks of implementation. We had the opportunity to comment on the pros and cons of the new way of working.”* (Nurse, ED1)

*“The education was limited to some basic information about the new triage system and a review of the warning signs in the WEST handbook.”* (Nurse, ED2)

*“We had a PowerPoint presentation lasting a maximum of one hour. Terrible! Those who were going to train us afterwards did not know the system themselves; if you had questions, there were no answers. You had to learn the system by yourself.”* (Nurse, ED2)

Educational shortcomings identified by ED2 staff included the lack of interactive, team-based formats such as simulation and tabletop exercises, as well as practical study materials. Participants requested case-based workshops, structured debriefings, joint sessions with physicians, bedside supervision during the initial phase, and systematic feedback. Although NIHSS and GCS were introduced within WEST, no dedicated training on these instruments was provided, leaving several nurses unfamiliar with their application.

### Organizational strategies

A majority of participants at ED1 agreed or strongly agreed that they felt prepared for the implementation of the new triage system compared with approximately one-third of participants at ED2 (82% vs. 32%).

*“In fact, the organization didn’t need to adapt, despite the absence of WEST in other hospitals and the ambulance services. We just transitioned from using RETTS© to WEST in our medical journals.”* (Nurse, ED1)

*“Perhaps it might not be ideal for nurses to be immediately thrown into triage duties. However, given the current circumstances, there may be no alternative.”* (Nurse, ED2)

### The system (performance of WEST)

**Fig 3:**
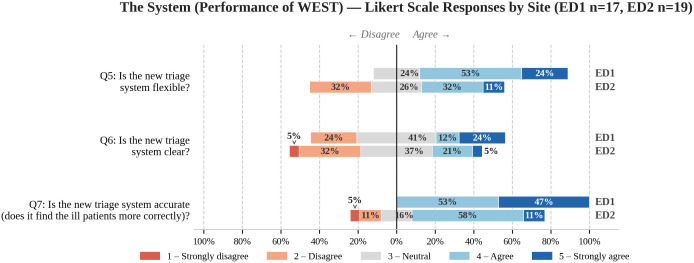
Responses related to performance of WEST.

Most participants agreed or strongly agreed that the new triage system was perceived as more flexible and more accurate than RETTS© in identifying severely ill patients (Fig 3). Despite these perceived advantages, a majority also agreed or strongly agreed that the new triage system was perceived as requiring greater clinical experience and was at times seen as more complex and less clear than RETTS©. Overall, participants at ED2 expressed less positive perceptions, reflected by consistently lower Likert ratings compared with ED1.

Distribution of Likert-scale responses (1–5) to questions related to the perceived performance of WEST at ED1 and ED2. Percentages may not sum to 100% due to rounding.

### Flexibility

A majority of participants agreed or strongly agreed that the new triage system was perceived as more flexible than RETTS©, with higher agreement at ED1 (76%) compared with ED2 (47%).

*“For me, the flexibility of the system is advantageous, especially with the pocket version, which is convenient to use bedside. However, it can pose challenges for new nurses, particularly those lacking experience in triage, as certain aspects of the process require clinical training. In contrast, RETTS© was foolproof-if you followed it mechanically, mistakes were unlikely.”* (Nurse, ED1)

*“Without prior experience, there are potential pitfalls when using WEST, making it potentially dangerous. However, with experienced staff who have undergone extensive training, WEST offers a simpler and more flexible alternative compared to RETTS©”* (Nurse, ED2)

While the flexibility enabled by clinical judgment was generally seen as an advantage, some participants noted that it placed higher demands on nurses with less clinical experience. Participants also highlighted the absence of an online version, gaps in the handbook, and lack of clarity regarding when to perform blood sampling.

### Clarity

Regarding clarity, approximately 40% of participants at each site perceived the new triage system to be at least as clear as RETTS©. At ED1, 35% agreed or strongly agreed that the new triage system was clearer, compared with 26% at ED2.

*“Being a new or recently graduated nurse and working in triage is not particularly easy. RETTS© proves to be simpler than WEST, especially for inexperienced individuals. The RETTS© binder served as a comprehensive guide, like a Bible, containing all the necessary information, including referrals.”* (Nurse, ED1)

*“At times, only a single word is used to describe a condition, like ‘ongoing bleeding,’ which lacks clarity. It becomes crucial to have additional support from the system, especially when you find yourself alone, to ensure clarity and understanding in such situations.”* (Nurse, ED2)

Participants also noted that WEST sometimes grouped conditions too broadly, for example, neurological symptoms, creating uncertainty. Unlike RETTS©, WEST did not provide a referral system, requiring nurses to rely on memory of parameters and risk factors.

### Accuracy

Of the 36 participants, 30 agreed or strongly agreed that the new triage system was perceived as more accurate than RETTS© in identifying severely ill patients, with complete agreement (100%) among ED1 respondents and 68% agreement at ED2.

*“When used correctly, WEST is more accurate compared to RETTS©. RETTS© tended to over-prioritize, whereas with WEST, patients categorized as red or orange were genuinely ill. The system’s reliability simplifies teamwork in the ED. Furthermore, WEST demonstrates improved prioritization for frail elderly individuals. While RETTS© may be more convenient for inexperienced nurses, WEST’s accuracy makes it a preferred choice for reliable patient assessments.”* (Nurse, ED1)

However, six participants disagreed, arguing that WEST was less precise.

*“WEST is less accurate, and you need experience to prioritize patients correctly. This lack of precision leads triage nurses to rely on their intuition rather than purely observational data. In contrast to RETTS©, where the orange triage level required monitoring, WEST extends monitoring to yellow and green priority levels. This results in an increased number of patients requiring monitoring, making it progressively challenging to maintain an overall overview.”* (Nurse, ED2)

### Driving forces (advantages of WEST)

**Fig 4:**
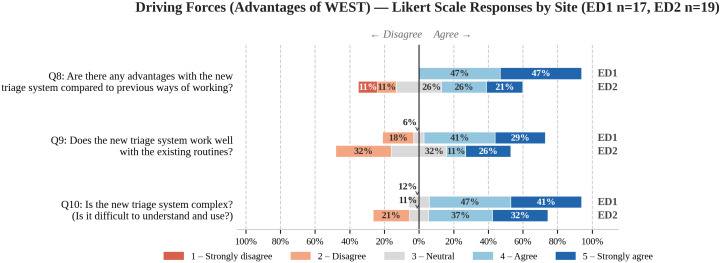
Responses related to driving forces for adoption of WEST.

Overall, participants agreed or strongly agreed that the new triage system performed well in terms of relative advantage, compatibility, and ease of use, though agreement rates at ED2 were consistently lower (Fig 4).

Distribution of Likert-scale responses related to perceived advantages and driving forces for adoption of WEST at ED1 and ED2. Percentages may not sum to 100% due to rounding.

### Relative advantage

Most participants agreed or strongly agreed that the new triage system had a perceived relative advantage over RETTS©, primarily attributed to a perceived reduction in assignment to the orange priority level and fewer unnecessary laboratory investigations. This perception was reported by 94% of participants at ED1 and 53% at ED2.

*“Fewer orange-prioritized patients result in fewer unnecessary blood samples (which is more cost-effective) and facilitate more accurate and easier identification of seriously ill patients.”* (Nurse, ED2)

Yet, participants also pointed to shortcomings, including vague descriptions of some warning symptoms and inconsistencies in blood sampling routines. They requested a more comprehensive handbook, including clearer referral guidance. Some noted that the provisional printed handbook was difficult to use in practice.

*“The pages of the pocket-version keep falling out, and I hope for improved quality.”* (Nurse, ED1)

### Compatibility

At ED1, 71% of participants agreed or strongly agreed that the new triage system was compatible with existing routines, compared with 37% at ED2.

*“The blood sampling routine in triage became more scaled down. Initially, this change led to longer waiting times due to inadequate guidelines recommending a minimum number of blood tests. However, presently, I find myself collecting more samples than WEST recommends.”* (Nurse, ED1)

Concerns were raised about WEST’s compatibility with established routines, particularly blood sampling for conditions such as deep vein thrombosis and subarachnoid hemorrhage.

### Ease of use

A majority of participants agreed or strongly agreed that the new triage system was easy to use, with higher agreement at ED1 (88%) compared with ED2 (68%). Nevertheless, several participants perceived that effective use of WEST required substantial clinical experience.

*“The system is straightforward and not difficult to follow, but it requires a lot from the nurse. There are no cheat sheets provided by the system, so it is crucial to have experienced personnel in triage.”* (Nurse, ED2)

Participants requested more detailed guidance, an online version for training, and improvements to the printed handbook to make it easier to navigate in practice.

### Overall comments

Across both sites, participant responses highlighted recurring themes related to training adequacy, laboratory routine compatibility, experience requirements, organizational readiness, and feedback mechanisms, all of which appeared to influence the adoption and clinical use of the new triage system. Several participants noted that the implementation coincided with the COVID-19 pandemic, which was described as contributing to limited training opportunities and increased workload during the transition period.

Participants consistently described the new system as more demanding and experience-dependent than RETTS©. Compatibility issues extended to patient handover and the transitional period during which ambulance services and neighboring EDs continued using RETTS©. Trialability was limited by simultaneous transition, and observability was weak, with insufficient feedback and debriefing reported. One individual involved in the implementation was specifically mentioned as accessible and responsive to feedback throughout the process.

*“With the new system, the majority of patients in the teams are green, creating a calmer atmosphere. It allows us to focus more on the yellow and orange priority levels. With RETTS©, the focus was on everyone, making it difficult to identify those who stood out.”* (Nurse, ED2)

*“Performing triage with WEST demands more from the individual due to the system’s lack of comprehensiveness. In some cases, additional explanations are necessary, requiring medical expertise to fully understand the process. Therefore, more experience is essential for effectively utilizing WEST.”* (Nurse, ED2)

*“Triage education becomes crucial, especially during system transitions. We need an online version for accessibility. Additionally, continuous education is essential, covering topics like chest pain with vegetative symptoms, NIHSS, NEWS2, and GCS.”* (Nurse, ED1)

*“If the new triage system had been introduced without changing the laboratory sampling routines at the same time, it would have been much easier to accept.”* (Nurse, ED2)

*“This new system requires anticipating what will be required; you have to think creatively. It demands experience but has significant potential.”* (Nurse, ED2)

Overall, WEST was perceived as promising but requiring further development, particularly regarding training, procedural adjustments, and educational support.

### Adoption-related attitude patterns

Adoption-related attitude patterns varied both within and between sites. ED1 showed a tentative tendency toward earlier adoption orientations, reflected in higher agreement across most Likert items and more frequently expressed confidence in the system. ED2 displayed a broader spread of responses, with more participants emphasizing the need for additional support and clearer guidelines before feeling fully confident. These patterns are interpretive rather than definitive, and individual variation was present at both sites. Selected quotes illustrating attitudes consistent with different adoption orientations are presented below.

### Innovators

*“I got what I needed to use the system. I was a superuser in the pilot study, which gave me practical training. Patients who are critically ill will be critically ill regardless of which triage system you use.”* (Nurse, ED1)

### Early adopters

*“Now, with NEWS and clinical judgment taken more seriously, when a patient is triaged as orange it truly means something.”* (Nurse, ED1)

### Early majority

*“I was hesitant at first. But we now differentiate patient priority more effectively with WEST, fewer patients are assigned to the orange level, and I experience that to be the case in practice. I can also make my own assessments and justify them in a different way.”* (Nurse, ED2)

### Late majority

*“They did not give good reasons. It was not implemented in consultation with those who work most in the emergency department.”* (Nurse, ED2)

### Laggard

*“I cannot see the advantages. The risks of missing patients due to insufficient experienced staff are real. Everything in healthcare should be clear and straightforward, WEST is not foolproof. Also, the reduction in laboratory sampling is negative. It causes delays in patient management in many cases, particularly for frail elderly patients with diffuse symptoms. I want to go back to RETTS©.”* (Nurse, ED2)

## Discussion

This study examined the early implementation of WEST in two EDs and demonstrated how local context shaped adoption. ED1, which served as the pilot site, reported more positive responses, while ED2 expressed mixed and sometimes critical views. Despite these differences, most participants across both sites perceived WEST as more accurate than RETTS© in identifying critically ill patients, with a perceived reduction in patients assigned to the orange priority level.

### Diffusion of Innovations as a sensitizing framework

Even though adopter categories cannot be definitively assigned, they can be interpreted tentatively based on patterns consistent with Rogers’ framework. From this perspective, there was a higher degree of attitudes resembling early adopters in ED1, while ED2 responses were more heterogeneous and included more cautious views resembling those associated with the early or late majority categories. Importantly, this does not imply that entire EDs can be assigned to a single category, rather, the data suggest relative tendencies within each local context.

Building on Rogers’ concepts of “Diffusion of Innovations” [21], the process of change can be understood as influenced by the norms, communication patterns, and structural characteristics of each social system. These contextual features appeared to shape how quickly and widely WEST was adopted in the two EDs. Differences in organizational culture and readiness likely contributed to this variation, though implementation maturity and timing may also have played a role. Furthermore, Moore’s notion of “crossing the chasm” illustrates the gap between enthusiastic early adopters (as observed in ED1) and more cautious groups who require evidence of clear benefits before embracing a new system (as seen in ED2) [27].

### Contextual explanations for site differences

Differences between ED1 and ED2 may reflect several interrelated contextual factors rather than fundamental differences in adopter characteristics. First, staff composition varied between sites: nurses at ED2 had, on average, more years in the profession, which may have shaped how they evaluated WEST. More experienced staff may have been more familiar with RETTS© and therefore more inclined to compare WEST against long-standing routines, or more accustomed to repeated organizational change. In contrast, the relatively less experienced cohort at ED1 may have perceived WEST as less disruptive and been more open to adoption, potentially reflecting novelty effects or differing baseline expectations. These experience-related factors could partly explain site variation in perceived clarity, ease of use, and reliance on clinical judgment.

Second, although training structure, staffing, and coverage were broadly similar across sites, differences in perceived preparedness and available support may reflect variation in local management engagement. At ED1, managers had been working actively to foster readiness for change and prepare staff for transition, which may have contributed to higher ratings of training sufficiency and perceived preparedness. At ED2, the shorter interval between implementation and data collection may have meant that managerial involvement was less visible to staff, influencing their perceptions of support. Third, ED2 implemented WEST during the COVID-19 pandemic, a period marked by unusually high workloads and concurrent organizational changes. These pressures may plausibly have reduced staff capacity to absorb new routines, so-called change fatigue, affecting how WEST was adopted during this critical period. ED1, as the pilot site, implemented WEST under comparatively stable conditions a year earlier. Fourth, the response rate of 46% and self-selected participation introduce a risk of self-selection bias, nurses with strong views toward the implementation may have been more likely to participate. However, participants represented a mix of senior and junior nurses across varying experience levels, suggesting the sample was not systematically skewed. In the absence of data on non-participants, we cannot fully exclude the possibility that the most engaged or most critical nurses were overrepresented. Nevertheless, data saturation was reached after approximately 20–25 interviews, with no major new themes emerging in the remaining interviews (n = 36), supporting the adequacy and depth of the data.

Finally, site differences in the data may partly reflect interviewer positionality. The first author was a resident physician at ED2 at the time of data collection, and participants at that site may have responded differently given familiarity with the interviewer, despite the voluntary and self-selected nature of recruitment. Several measures were taken to mitigate this influence, including strict adherence to a standardized interview guide, structured field notetaking, and investigator triangulation during analysis, as described in the Methods. Nevertheless, the potential for site-differential response bias cannot be fully excluded and should be considered when interpreting differences between sites. Direct comparison of ED1 and ED2 is therefore problematic, as a *ceteris paribus* condition (“all else equal”) could not be assumed. The findings should be understood as descriptive, focusing on ‌‌how local contexts shaped adoption at different stages of implementation.

### Clinical judgment and novice usability

A defining feature of WEST is its reliance on nurses’ clinical judgment alongside structured parameters. Participants generally valued this flexibility, particularly for identifying frail elderly patients, but several expressed concern that the new system may be more challenging for less experienced nurses. In contrast to RETTS©, which provided more prescriptive protocols, WEST placed greater demands on prior knowledge and contextual experience. These views should be understood primarily as perceptions reported by participants rather than conclusions directly linked to measured experience levels in the dataset. Prior literature suggests that clinical judgment can both enhance acuity discrimination and introduce variability, among novice practitioners, and that senior nurses may under-triage while junior nurses may over-triage, complicating staffing strategies [38,39]. This aligns with prior implementation research showing that innovations placing greater demands on individual judgment tend to be more challenging to adopt in settings where staff experience varies widely [17,19]. In addition, perceptions of preparedness and competence may be influenced by cognitive mechanisms such as overconfidence among less experienced staff or reduced feedback loops during early implementation. This represents a potential limitation of self‑reported data, as responses may partly reflect subjective confidence rather than objective performance. These findings therefore highlight the perceived need for structured support, mentorship and feedback opportunities to ensure that the flexibility of WEST is balanced with consistency and safety.

### Educational and organizational implications

Although the educational interventions were designed and delivered similarly at both sites, participants’ perceptions differed markedly. Nurses at ED1 described the education as sufficient, interactive, and supported by supervision, while those at ED2 perceived it as brief, superficial, and lacking practical guidance. This discrepancy highlights how staff perceptions of preparedness may diverge even when formal training structures are equivalent.

Participants consistently emphasized that WEST required more than short information sessions for safe and effective use. They called for simulation-based training, bedside supervision, structured case reviews with physicians, and explicit instruction in NIHSS and GCS. The lack of a comprehensive handbook and the absence of a digital version were identified as barriers. These findings align with prior literature recommending experiential learning, continuous feedback, and accessible educational tools for complex system changes [40].

Organizational readiness also proved critical. ED2’s experience in the present study may reflect a pattern identified in prior research, whereby subcultures within healthcare settings tend to resist change when implementation strategies do not align with existing cultural norms [18]. ED2’s experience illustrated how simultaneous reforms, such as pandemic-related adaptations, undermined implementation even when staff acknowledged WEST’s clinical advantages. Aligning the timing of major changes with organizational capacity is therefore essential to minimize disruption.

### Timeliness

Although data for this study were collected in 2021, the implementation of the WEST triage system across the Western region occurred gradually between 2019 and 2023, and the system is currently being prepared for adoption in additional regions. While time-sensitive aspects relate primarily to the system’s clinical performance, the determinants examined here, such as organizational readiness, education and training, and staff motivation, represent fundamental components of implementation processes that remain relevant regardless of when implementation occurs.

### Summary of implications

Taken together, these findings suggest that successful implementation of WEST was shaped less by the intrinsic qualities of the triage system than by the organizational context in which it was introduced. Adoption appears to be influenced by communication, motivation, and training strategies, as well as the ability of each ED to manage competing changes. While WEST was widely perceived as improving triage accuracy, challenges with clarity, novice usability, and contextual readiness underscore the need for carefully staged and well-supported rollouts. Viewed through Rogers’ diffusion framework, these contextual differences highlight how social system characteristics shape diffusion, while Moore’s “chasm” reminds us that enthusiasm among early adopters does not automatically translate into broader uptake without demonstrable advantages for more cautious groups [27]. In this case, WEST has since expanded substantially across Swedish EDs, yet this study underscores that innovations face risks during their initial phases of dissemination.

While these findings are grounded in the Swedish emergency care context, several implementation determinants, including organizational readiness, management engagement, and training quality are likely transferable to other settings. However, Sweden’s tax-funded healthcare system, in which EDs receive both referred and self-presenting patients, alongside the specific transition from RETTS© and pandemic timing, may limit direct comparability with healthcare systems operating under different funding models or referral structures.

### Limitations

This two-site design, with ED1 as the pilot and asynchronous implementation at ED2 during COVID-19 limited comparability and precluded causal inference. The interval between implementation and data collection differed substantially across sites, interviews were conducted 17 months after implementation of WEST at ED1 and 6 months after implementation at ED2. This discrepancy reflects real-world conditions rather than a planned design choice, as these were the only EDs in the region using WEST at the time, and the region chose to review early outcomes before wider rollout. While this temporal difference may have influenced recall, it also provided complementary insight by capturing both early and more established implementation phases. Baseline performance differences between sites may further have influenced staff perceptions, and external validity was constrained by the regional, two-site design.

A key methodological limitation concerns data collection. The interviews were documented through detailed real‑time notes rather than audio recordings, which may have limited the capture of nuance, potentially introduced selective recording, and increased the risk that reconstructed quotes do not fully reflect participants’ exact phrasing. Without audio recordings, subtle features such as hesitation, uncertainty, contradiction, emotional tone, and self-correction were not available for analysis. These qualities may be particularly relevant in a study where attitudes toward implementation are central, as responses that were uncertain or conflicted may carry different meaning than their written form suggests. Given that the present study primarily aimed to explore attitudes shaping the adoption and diffusion of the implementation, structured field notes were considered appropriate for capturing the relevant content. To mitigate potential limitations associated with this approach, data collection involved detailed systematic note-taking procedures and participant verification through member checking, but the absence of verbatim transcripts may still affect analytic depth and qualitative rigor.

Finally, assessments of triage outcomes, including reductions in over-triage, were based on participants’ perceptions rather than objective measurements. Future studies should incorporate quantitative outcome data to validate these findings.

### Interviewer positionality and reflexivity

All interviews were conducted by the first author, a resident physician in emergency medicine at ED2 at the time of data collection, with clinical experience but no formal leadership responsibilities at either site. This dual position as both clinician and researcher may have influenced how openly some participants responded, particularly at ED2, where an established working context existed. Participants at ED1 did not share this relationship with the interviewer, though awareness of the interviewer’s role within the regional emergency care system may still have shaped interactions to some extent.

Several measures were taken to minimize potential bias. Recruitment was entirely self-selected, with nurses initiating contact voluntarily, reducing the likelihood of perceived hierarchical pressure. A standardized interview guide was followed strictly across both sites, and a structured note-taking approach ensured consistency. During analysis, rigor was strengthened through investigator triangulation, collaborative coding, member checking, and the use of an explicit codebook, with within-site analyses conducted prior to cross-case comparison. Nevertheless, some degree of positionality-related influence on data generation and interpretation cannot be fully excluded.

## Conclusions

Most nurses perceived WEST as more accurate than RETTS© for identifying critically ill patients and reducing over-triage. The implementation process, however, highlighted how adoption was shaped by local context, particularly communication, motivation, education, and organizational readiness. Treating each ED as a distinct social system and applying “Diffusion of Innovations” as a conceptual lens helped explain variation in adoption patterns. These findings suggest that future implementations of new triage systems may benefit from being carefully staged, adequately resourced, and aligned with other organizational changes to minimize disruption.

## Supporting information

S1 File Interview guide. Questions as they were asked by the questioner.
(PDF)

S2 File Informed consent. English translation of the informed consent.
(DOCX)

S3 File COREQ Checklist. COnsolidated criteria for REporting Qualitative research (COREQ) completed checklist.
(PDF)

## Abbreviations

ED: Emergency Department
IQR: Interquartile Range
GCS: Glasgow Coma Scale
NEWS2: National Early Warning Score 2
NIHSS: National Institutes of Health Stroke Scale
RETTS©: Rapid Emergency Triage and Treatment System
COREQ: Consolidated criteria for Reporting Qualitative research
WEST: West coast system for triage.
