# Supplementary material for: Evaluation of the implementation process of a new emergency triage system: West coast system for triage (WEST)
Source: PLoS One. 2026 Jun 11;21(6):e0350323. doi: 10.1371/journal.pone.0350323 (PMC13258005; doi:10.1371/journal.pone.0350323)
Supplement: S1 File — Questions as they were asked by the questioner. (PDF) [file pone.0350323.s001.pdf]

Questionnaire, questions as they were asked by the questioner

Questions

The Change (Implementation of WEST) (Jansen et al., 2011)

Q1: Information strategies: Have you received enough information about the new triage system?

Q2: Motivation and consensus strategies: Have you got good reasons why you change from one triage system to another? (From those who informed you) Is it clear why it's good?

Q3: Educational strategies: Have you received good and sufficient education about the new triage system? Something you wanted?
Q4: Organisational strategies: Is the organization (the emergency department as a workplace) adapted to the new triage system? 5 does not need to adapt, 1 needs much adaptation

The system (Performance of WEST) (compared with RETTS) (Cicolo et al. 2020)

Q5: Flexibility: Is the new triage system flexible

Q6: Clarity: Is the new triage system clear
Q7: Accuracy: Is the new triage system accurate (does it find the sick patients more correctly?)

Driving forces (Pros/Cons of WEST(Sutton et al, 2020)

Q8: Advantage: Are there any advantages with the new system compared to previous ways of working?

Q9: Compatibility: Does the new system work well with the exciting routines, or did you have to change a lot of other things?

Q10: Complexity: Is the new triage system complex? (Is it difficult to understand and use?)

Overall comments
