# Supplementary material for: Evaluation of the implementation process of a new emergency triage system: West coast system for triage (WEST)
Source: PLoS One. 2026 Jun 11;21(6):e0350323. doi: 10.1371/journal.pone.0350323 (PMC13258005; doi:10.1371/journal.pone.0350323)
Supplement: S2 File — English translation of the informed consent. (DOCX) [file pone.0350323.s002.docx]

**WEST — Perceived Benefits and Implementation of a New Triage System**

**Participant Information**

We would like to ask whether you are willing to participate in a research project. In this document you will find information about the project.

| **1. Background and Purpose** | It is well established that triage in emergency departments is a challenge both in identifying the most acutely ill patients and in avoiding contributions to increasing crowding. With this sub-study we aim to: 1) obtain your perception of the strengths and weaknesses of WEST, and 2) gather information about how you perceive the implementation of WEST at your workplace. The principal investigator is Västra Götalandsregionen, Regionens hus, SE-462 80 Vänersborg. |
| --- | --- |

| **2. Participants** | We are asking physicians and nurses who perform triage in emergency departments. You are being approached because you are best placed to describe the perceived benefits of WEST and to describe the implementation process. |
| --- | --- |

| **3. How is the Study Conducted?** | The interview is individual and is expected to last a maximum of 45 minutes, conducted near your workplace. Detailed written notes will be taken during the interview to allow analysis of the material afterwards. |
| --- | --- |

| **4. What are the Risks?** | Participation in the study is not considered to be associated with any risks for you as a participant. Consideration will naturally be given if any person experiences discomfort when discussing problematic situations in healthcare. Participants share only what they themselves wish to share. |
| --- | --- |

| **5. Are There Any Benefits?** | Through the study, you contribute to ongoing long-term research on emergency departments related to the introduction of new models and specifically the outcomes of WEST. |
| --- | --- |

| **6. Data Handling and Confidentiality** | Full confidentiality is guaranteed and data will be stored and handled in accordance with Swedish regulations and the legal basis of the EU General Data Protection Regulation. Published data will not be traceable to any individual. Your responses will be handled so that unauthorised persons cannot access them and will be stored securely. |
| --- | --- |

**WEST — Perceived Benefits and Implementation of a New Triage System**

**Participant Information**

| **7. How Will I Receive Information About the Study Results?** | Study data will be stored for 10 years to allow for verification. The Executive Board of Sahlgrenska University Hospital is responsible for the processing of personal data. In accordance with the Personal Data Act (1998:204), you have the right to request information about which personal data are being processed. You may submit your request to the contact person below or to Sahlgrenska University Hospital, Data Protection Officer Susan Lindahl, SE-413 45 Gothenburg. Telephone: +46 31-343 27 15. Publication in a scientific journal is planned, as well as inclusion in a doctoral thesis. |
| --- | --- |

| **8. Compensation and Insurance** | Participation in the research project is unpaid, takes place during working hours, and is covered by the insurance included in your employment at Sahlgrenska University Hospital. |
| --- | --- |

| **9. Voluntary Participation** | Participation in the study is entirely voluntary and you may withdraw your consent at any time, without providing any explanation. |
| --- | --- |

| **10. Responsible Researchers** | **Professor Eric Carlström**  Department of Health and Care Sciences, University of Gothenburg  GEMREG, Sahlgrenska University Hospital  eric.carlstrom@gu.se \| +46 70-273 81 26  **MD Samah Habbouche**  Emergency Department, GEMREG, Sahlgrenska University Hospital  samah.habbouche@vgregion.se \| +46 76-138 73 33  **MD PhD Lina Holmqvist**  Head of Section, Senior Physician, Emergency Department, GEMREG  Sahlgrenska University Hospital / Emergency Development Centre SU  Department of Medicine, Sahlgrenska Academy / University of Gothenburg  lina.holmqvist@vgregion.se \| +46 31-342 88 75  **MD Tobias Carlson**  Head of Section, Senior Physician, Emergency Department  Sahlgrenska University Hospital  Head of Emergency Development Centre SU  tobias.carlson@vgregion.se \| +46 72-515 39 62 |
| --- | --- |

**WEST — Perceived Benefits and Implementation of a New Triage System**

**Participant Information**

**Consent to Participate in the Study**

I have received written and verbal information about the study and have had the opportunity to ask questions.

I am entitled to keep a copy of this written information.

☐ I consent to participate in the study: **WEST — Perceived Benefits and Implementation of a New Triage System**

☐ I consent to my personal data being processed in the manner described in the participant information.

Gothenburg, Date:

Name:
