# Supplementary material for: Evaluation of the implementation process of a new emergency triage system: West coast system for triage (WEST)
Source: PLoS One. 2026 Jun 11;21(6):e0350323. doi: 10.1371/journal.pone.0350323 (PMC13258005; doi:10.1371/journal.pone.0350323)
Supplement: Attachment — Submitted filename: Evaluation of of WEEST coast System.docx [file pone.0350323.s004.docx]

**Reviewer comments**

Manuscript Number: PONE-D-26-01867

Article Type: Research Article

Full Title: Evaluation of the implementation process of a new emergency triage system: WEST coast System for Triage (WEST)

Short Title: Evaluation of WEST Emergency Triage System Implementation

**Abstract:** - **comments**

**Paragraph**: In western Sweden, several EDs transitioned from the Rapid Emergency Triage and Treatment System (RETTS©) to the WEst coast System for Triage (WEST), based on principles of the South African Triage Scale

**Paragraph:** While WEST was introduced and spread rapidly, reports suggested challenges during implementation.

**Objective**: This study mapped the implementation process at the first two EDs (ED1 and ED2) adopting WEST and identified barriers and facilitators. We focused on the transition from RETTS© to WEST and explored staff experiences across implementation stages.

**Methods:** We conducted semi-structured individual interviews with 36 triage nurses from two hospitals (ED1 n=17; ED2 n=19). Interviews explored experiences with WEST, implementation challenges, and comparisons with RETTS©. Rogers’ “Diffusion of Innovations” was used as a sensitizing framework, and each ED was treated as a distinct social system. Descriptive analyses of Likert-scale items complemented the qualitative analysis.

**Results**: ED1 participants expressed greater enthusiasm and perceived WEST as a notable improvement. ED2 responses were more varied, with reservations commonly targeting the implementation process and concurrent organizational changes. Across both sites, most nurses (30/36) perceived WEST as more accurate than RETTS© in identifying critically ill patients. Conclusions Implementing WEST was feasible but context dependent. Information, motivation and consensus-building, education, and organizational readiness emerged as important factors in the implementation process. Treating each ED as a separate social system and using “Diffusion of Innovations” conceptually helped explain adoption heterogeneity.

**Background**

Well-articulated with clear theoretical framework of ‘diffusion innovation’

**Paragraph:** However, few studies provide in-depth investigations of health professionals' reactions to implementation in an ED context, (17, 26) and no previous studies have investigated health professionals’ responses to introducing new triage system in Sweden. One study from a Danish ED explored what happened when health professionals faced the implementation of a new policy that changed their organization

**AIM:**

The aims/ objectives are SMART, however the last statement is vague and does not constitute a specific objective. ‘’*We also compared implementation 112 processes between ED1 and ED2’’.*

The study had two aims: (i) to explore how triage nurses experienced the transition from 110 RETTS© to WEST, and (ii) to describe perceived benefits and drawbacks of WEST relative to 111 RETTS© regarding flexibility, clarity, and accuracy. We also compared implementation 112 processes between ED1 and ED2.

**Materials and Methods:**

***Study context****:* study area is well described, clearly demonstrating the need to collection from this area, however this last statement creates additional research question or appears to introduce a new objective, effectively shifting your study toward a comparative design. *‘’ED2 had longer length-of-stay and time-to-physician than ED1’’*

***Study population and data collection:*** *qualitative data collection well done, however, there no clearly sampling frame work to recruit the participants and the period of data collection appears very short. This ED centers seems huge with high number of nurses and thus collection of data should at least take one month or more.*

***Study design and methodology:*** *The study design is accurate and validity of study is well demonstrated, however the researcher should try and avoid the word the ‘we’*

***We used semi-structured interviews informed by a literature review and prior studies. The 149 interview guide drew on “Diffusion of Innovations” to frame attitudes to innovation diffusion and 150 included Likert-scale items to gauge agreement.***

***Data analysis:*** *well done. Credibility of the data analysis was strengthened by use of two independent researchers who took the detailed notes.*

***Ethical considerations:*** *while the study followed the Declaration of Helsinki and was approved by the 176 Regional Ethical Review Board in Gothenburg (Dnr1078-18; 2020-06571), there is no clear explanation of information entailed in the informed consent.*

***Suggestion****: A sample copy of the informed consent should be provided.*

***Results:*** *Characteristics of the study population is shown clearly, Likert-scale was used toe summaries, the ED1 respondents which was reported as positive perceptions of WEST. Differences in the process of implementing WEST was also reported and responses of the participants was captured well.* However, the Discussion section requires detailed comparison with previous studies that reported either similar findings or contradictory results.

This study has minor corrections.

Thank you
