# Supplementary material for: Evaluation of the implementation process of a new emergency triage system: West coast system for triage (WEST)
Source: PLoS One. 2026 Jun 11;21(6):e0350323. doi: 10.1371/journal.pone.0350323 (PMC13258005; doi:10.1371/journal.pone.0350323)
Supplement: Attachment — Submitted filename: Reviewer suggestions.docx [file pone.0350323.s005.docx]

**Reviewer Suggestion**

**Title: Evaluation of the implementation process of a new emergency triage system: WEst coast System for Triage (WEST)**

The current manuscript addresses a very important and relevant study addressing implementation of triage innovation in the context of emergency department. The research question is clinically relevant, the two-site comparison is a strength mixed approach is appropriate.

Few concerns suggested are related to methodological rigor, adherence to qualitative reporting guidelines, data availability, and some instances where conclusions may exceed what the data support.

**1.Data Saturation Not Addressed**

The manuscript does not address data saturation, which is a key consideration in qualitative research. For transparency and methodological rigor, the authors should clarify whether saturation was achieved and how this was determined.

**2. Regarding the researcher (line 141):** The first author is a resident physician in emergency medicine at ED2 at the time of data collection While the authors mention this in the limitations (lines 462–464), the discussion is insufficient.

**3. Data availability**: Data Availability Does Not Meet the journal requirement.

**4. Application of Rogers' Framework Lacks Systematic Rigor**

Line 81: Rogers' Diffusion of Innovations is described as a 'sensitizing framework' (line 81), which is appropriate for a qualitative study. However, the manuscript assigns adopter categories to participants and EDs without describing how these classifications were determined. No validated instrument or systematic criteria were used for this categorization.

Modify Discussion that implies definitive adopter categorization (e.g., lines 383–385).

**Minor Modifications:**

**5. Ethics Approval Number Discrepancy**

Two different sets of ethics approval numbers appear in the manuscript. Line 177 states: 'Dnr1078-18; 2020-06571' while the Declarations section (line 482) states: 'Dnr 2020-06571; 2021-0209.' This discrepancy must be resolved. Please clarify whether both refer to the same study or if there were separate approvals for different phases.

**6. Inter-Rater Reliability Not Quantified**

The authors describe independent coding by two researchers followed by reconciliation (lines 166–168). However, no quantitative measure of inter-rater agreement is reported. While this is not always required in deductive qualitative analysis, or an explicit justification for its absence is to be mentioned

**7. Overclaiming in Conclusions**

Several statements in the Discussion and Conclusions imply causal relationships that the study design cannot support:

- Line 404–406: The claim that COVID-19 'caused change fatigue' is an interpretation, not an established finding. Rephrase as a plausible contributing factor.
- Line 440–441: 'Adoption depended on communication, motivation, and training strategies' implies causality. Suggestion is to rephrase to 'adoption appeared to be influenced by...
- Line 473: 'Future implementations should be carefully staged, adequately resourced, and aligned' - this is a strong prescriptive recommendation from a two-site qualitative study. Consider rephrasing 'these findings suggest that future implementations may benefit from...'

**8. Sampling method**

The Methods section states that nurses were invited via email and workplace meetings (line 139), but does not specify the sampling method. Was this convenience sampling, purposive sampling, or total population sampling? The inclusion criteria are stated (line 138), but the approach to recruiting the sample should be named and justified

**9. Participant Anonymity Concerns**

The study names the specific hospital (Sahlgrenska University Hospital) and identifies participants by ED site. Given the relatively small sample (17 and 19 nurses respectively), and the use of the specific site as EDs, there is a risk of participant identification, particularly for male nurses (5 per site). The authors should confirm that steps were taken to protect anonymity beyond removing names and consider whether any quotes could be identifying.

10. **Reference Formatting**

Reference 39 appears incomplete (line 603 '). Ensure all references include DOIs where available and also several references do not meet the style of referncing.

Conclusion: MANUSCRIPT STRENGTHS

The following aspects of the manuscript are commended:

1. The two-site comparative design provides valuable insight into the implementation outcomes.
2. The use of Rogers' Diffusion of Innovations as a sensitizing framework is well-suited to the research question and adds theoretical depth.
3. The mixed approach combining qualitative interviews with Likert-scale items provides complementary perspectives on the implementation experience.
4. The clinical relevance of the topic is high — understanding implementation barriers and facilitators for triage systems has direct implications for patient safety and ED management.
