# Supplementary material for: Evaluation of the implementation process of a new emergency triage system: West coast system for triage (WEST)
Source: PLoS One. 2026 Jun 11;21(6):e0350323. doi: 10.1371/journal.pone.0350323 (PMC13258005; doi:10.1371/journal.pone.0350323)
Supplement: Attachment — Submitted filename: Response_to_reviewers.docx [file pone.0350323.s006.docx]

**Response to reviewers**

Dear Editor,

We would like to express our sincere gratitude to the Academic Editor and the eight reviewers for the opportunity to revise our manuscript. Your comments were thoughtful and constructive and have meaningfully strengthened the work. In the interest of readability, we have not expressed our gratitude in every answer for readability but certainly we are thankful for every interesting and developing comment. Also, we have organized our responses thematically rather than addressing each comment individually. We hope this approach is acceptable and remain happy to address any further questions or comments.

Sincerely,

The Authors

**Journal Requirements** 
 
The following actions have been performed in response to journal requirements: 

- The manuscript has been revised to meet the criteria of PLOS ONE.
- All ethical approval numbers listed in the manuscript and submission metadata have been corrected and are now consistent across all documents.
- The financial disclosure has been updated according to the journal's suggestion.
- All funding-related text has been removed from the manuscript.
- The data availability statement has been modified to meet journal requirements.
- The ethics statement now appears only in the Methods section.
- The manuscript has been revised so that Table 1 is referenced correctly.
- We thank the journal for the recommendation regarding citations.

Where multiple reviewers raise the same issue, all are noted in 'Reviewer(s)' column.

**Abstract**

| **Section** | **Comment** | **Reviewer(s)** | **Action taken** |
| --- | --- | --- | --- |
| Abstract | "WEst" → "WEST" correct formatting, avoid mixed capitalisation throughout | R4 | Revised to meet journal requirement. |
| Abstract | Abstract focuses only on challenges - add mention of potential opportunities with implementation | R4 | Sentence added Line: 19 |
| Abstract | Remove "we" -replace with "The study focused…" | R4 | “We” have been changed throughout the manuscript, lines: 22, 24 |
| Abstract | Aim is SMART but wordy - reconstruct more concisely | R4 | Aim is reconstructed as suggested, lines: Line 21-23 |
| Abstract | Avoid "we"/"I" throughout - use academic language e.g. "The study conducted semi-structured individual interviews" | R4, R5 | “We” have been changed throughout the manuscript, lines: 22, 24, |
| Abstract | Missing: ethical considerations, sampling procedure, data analysis method - include all research processes in abstract | R4 | Revised, Lines 24, 27-31 |

**Background / Introduction**

| **Section** | **Comment** | **Reviewer(s)** | **Action taken** |
| --- | --- | --- | --- |
| Background | Timeliness/novelty: data nearly 5 years old - justify ongoing relevance or frame explicitly as implementation case study with enduring lessons; add Discussion paragraph on continued relevance | R1 | Revised in discussion section lines: 512-519 |
| Background | Add linkage between acuity assignment and over-triage - how does acuity relate to over-triage? | R3 | Linkage has been added as suggested. Line: 57-59 |
| Background | Add transitional sentence between RETTS/WEST and organisational culture (example provided) | R3 | Transitional sentence has been added as recommended. Lines 70-74 |
| Background | Lines 88–89: revise Rogers/EDs wording to e.g. "Rogers' concepts are particularly useful in EDs because EDs function as unique social…" | R3 | Has been revised as suggested: Lines 94-97 |
| Background | Lines 96/97: clarify Rogers→Moore transition; add sentence introducing 'Crossing the Chasm' concept | R3 | Sentence has been added as suggested: Lines 100-103 |
| Background | Conclude framework section by tying back to study design; if 'Crossing the Chasm' not used in design, move to Discussion | R3 | A concluding sentence has been added, and a clarification has been included. Lines 108-110 |
| Background | Change 'accuracy' to 'perceived accuracy' in aims | R3, R1 | Changed as recommended. Line 118 |
| Background | Minor grammatical corrections (7 items: tense, prepositions, articles, comma, plural) | R3 | All corrected as suggested |
| Background | Strengthen link to Danish ED study referenced in background paragraph | R4 | Sentence added as suggested. Lines 85-87. |
| Background | General readability/language issues; Rogers/DoI utility questioned; 'frontline nurse' → 'triage nurse'; Line 61: spell out NEWS2; references at end of sentences throughout | R5 | Following lines have been revised for readability: Triage nurses: 51 NEWS2-Line 66  Rogers DOI: 89-102 |

**Aims**

| **Section** | **Comment** | **Reviewer(s)** | **Action taken** |
| --- | --- | --- | --- |
| Aims | Last aim statement is vague — reframe as a secondary aim or integrate more explicitly | R4, R3 | A secondary aim is added. Lines 118-119 |

**Methods**

| **Section** | **Comment** | **Reviewer(s)** | **Action taken** |
| --- | --- | --- | --- |
| Methods | No audio recordings: use correct terminology ('detailed interview notes'); describe note-taking process; clarify what 'cleaning' entailed; temper evidentiary weight of quotes | R1, R6, R7 | Revised for transparency, section data collection. Lines 170-187 |
| Methods | Interviewer positionality: add dedicated reflexivity subsection covering relationship to participants, power dynamics, and mitigation steps | R1, R7, R8 | Added sections in the background and discussion. Lines: 188-199 and 562-578 |
| Methods | Data saturation not addressed - clarify whether saturation was achieved and how it was determined | R8 | Revised as suggested. Lines 181-186 |
| Methods | Sampling method not named - specify whether convenience, purposive, or total population sampling and justify | R4, R8 | Sampling method added. Lines 166-167 |
| Methods | Soften HTA claim: change to 'effective in supporting patient prioritization and ED flow under crowded conditions' | R3 | Revised as suggested. Lines 145-146 |
| Methods | Provide source for ED2 longer LOS/time-to-physician; if from HTA, move to that paragraph | R3, R4 | Source added. Lines 159-160 |
| Methods | Line 123: clarify 'in this study' - HTA or the present study? | R3 | Clarified in line 146 |
| Methods | Reframe paragraph from line 125; remove stroke scale and coma scale | R3 | Reframed as suggested, stroke and Glasgow coma scale are retained as they reappear in a quote. |
| Methods | Clarify 'pocket-sized version' → 'pocket-sized WEST reference card' | R3 | Clarified as suggested. Line 157 |
| Methods | Provide more detail on transcription: who transcribed, how (typed/handwritten), how quotes were handled | R3, R7 | Added in data collection section. Lines 171-180 |
| Methods | Were there any missing responses? If so, how were they handled? | R3 | Added as suggested. Lines 202-204 |
| Methods | Line 158: expand on how unexpected themes were identified and grouped | R3 | Added as suggested. Lines 135-136 |
| Methods | Minor grammatical corrections (2 items): hospital size phrasing; 'cleaned of pauses' → 'edited for readability while preserving wording' | R3 | Revised as suggested |
| Methods | State study design as first sentence in Methods; avoid personal pronouns; complete COREQ checklist and include as appendix; Table 1: use 'interquartile range' not 'range' | R5 | All revised as suggested. Study design: Line: 124  COREQ: Lines 185-187  Revised IQR: Lines 225-228 |
| Methods | Add statement that no inferential statistical testing was planned or performed | R7 | Added as suggested. Lines 204-205. |
| Methods | Inter-rater reliability not quantified - report a measure or explicitly justify absence | R8 | Added as suggested. Lines: 211-213 |
| Methods | Data collection period appears very short given size of ED; provide informed consent sample | R4 | Revised and added as supplement. Line 169 |
| Methods | Ensure ethics approval identifiers are consistent throughout manuscript | R1, R8 | All revised |
| Methods | Mixed-methods clarity: integrate Likert component more formally or position as supportive descriptive context | R1 | Revised under study design and methodology. Lines 123-136. An throughout the result section. |
| Methods | Provide clearer detail on coding procedures: portion double-coded, codebook development, handling disagreements, negative cases | R1 | Revised in the data analysis section. Lines 200-214 |

**Results**

| **Section** | **Comment** | **Reviewer(s)** | **Action taken** |
| --- | --- | --- | --- |
| Results | Add response rate: 36 of 78 eligible nurses participated (response rate 46%; ED1: 17/44, ED2: 19/34) | R3 | Response rate added as suggested. Line 224. |
| Results | Table 1: differences in professional experience not integrated as explanatory variable for site differences | R1 | Revised and added in the discussion section. Lines 452-459 |
| Results | 'Accuracy' claims not supported by objective evidence — reword to 'perceived accuracy' throughout; clarify how participants operationalised 'accuracy' | R1, R3 | Revised to perceived accuracy. Lines 120, 130, 234, 527 |
| Results | ED1 higher Likert ratings: discuss staff mix, seniority, and implementation maturity as alternative explanations | R1 | Revised in discussion section Contextual explanations for site differences. Lines 450-459 |
| Results | Training/preparedness large site differences (76% vs 32%; 82% vs 32%) - provide deeper contextual explanation | R1 | Revised in discussion section Contextual explanations for site differences. Lines 460-470 |
| Results | Clinical judgment, experience, and possible cognitive bias - present primarily as perceived risk (lines 281, 347, 357) | R1 | Revised in discussion section Clinical judgment and novice usability. Lines 492-495 |
| Results | Incomplete Likert reporting (line 287 and similar) - report complete distributions for each item or provide in supplementary material | R1 | Figures and likert reporting in the result have been revised for readability. |
| Results | 'Over-triage' language potentially misleading - replace with 'reduced assignment to orange priority level' | R1, R3 | Language revised as suggested |
| Results | Experience-dependent triage as operational risk - discuss in Results and Discussion (lines 347, 357) | R1 | Discussed in discussion. Lines 489-492 |
| Results | Line 190: reword Q7 reference to be self-explanatory for readers without the questionnaire | R3 | Reworded as suggested. Lines 234-235 |
| Results | Use consistent Likert wording throughout: 'agreed or strongly agreed' (2 examples given) | R3 | Revised throughout the result section as recommended. |
| Results | Add dedicated Adoption Patterns section: early vs late adopter characteristics by site | R3 | Added adoption pattern as suggested. Lines 422-428 |
| Results | Minor grammatical corrections (5 items): 'were older'; 'longer tenure'; gender phrasing; 'of the education'; 'perceived WEST to be' | R3 | Revised as suggested. |
| Results | Add cross-site summary at the end of each results section | R3 | We acknowledge this suggestion. However, given the extensive cross-site comparisons already embedded within each section, and the overall cross-site summary provided in the Overall comments section, we felt that adding additional summaries would be repetitive rather than informative. We have therefore chosen not to implement this change. |
| Results | Put Likert percentages in figures in addition to mentioning them in text | R5 | Figures revised for readability |

**Discussion**

| **Section** | **Comment** | **Reviewer(s)** | **Action taken** |
| --- | --- | --- | --- |
| Discussion | Cross-site comparison heavily confounded - reframe ED1 vs ED2 differences as context-dependent and exploratory; treat implementation maturity and COVID-19 as plausible primary drivers | R1 | Revised Lines: 450-459, 466-470, see also revised subsection “Contextual explanations for site differences” |
| Discussion | Management involvement: consider more suitable theory (e.g. CFIR, NPT) or strengthen justification; explain how manager/staff discrepancies were handled analytically | R1 | Revised Lines: 460-470, |
| Discussion | Rogers' framework lacks systematic rigor - adopter categorisation without validated instrument; modify lines 383–385 to avoid implying definitive categorisation | R8 | Discussion revised. Lines 435-449 |
| Discussion | Further link findings to Rogers' specific attributes: relative advantage, complexity, compatibility, communication channels | R3 | Due to conflicting reviewer feedback regarding the use of Rogers' Diffusion of Innovations framework, we have chosen to retain the current level of theoretical discussion rather than expanding it further. Beyond the additions made to the Adoption-related attitude patterns section, we did not find it appropriate to strengthen or elaborate the theoretical connections further at this stage |
| Discussion | Add reference for WEST expansion (line 446); clarify what 'early setback' refers to | R3 | There is no published study that confirms this. |
| Discussion | Line 449: make clear this study underscores risks during initial phases of dissemination | R3 | Added as recommended. Line 532-534 |
| Discussion | Remove repetition of 'training designed similarly but perceived differently' (lines ~399–403 and ~421–425); use freed space to add new content e.g. presence of champions | R3 | Removed as suggested. |
| Discussion | Line 392: change 'demonstrated faster and more positive adoption' to 'showing more rapid and positive adoption' | R3 | Sentence removed |
| Discussion | Did ED1 participants know the interviewer? Discuss as potential bias source | R3 | Revised under interviewer positionality and in the discission under interviewer reflexivity. Lines 569-578 |
| Discussion | Discussion lacks comparison with previous studies reporting similar or contradictory findings | R4 | Comparisons with previous studies have been added in the Discussion section. Specifically, references to Stefansdottir et al. [17], Nilsen et al. [19], and Carlstrom and Olsson [18] have been incorporated to contextualise findings related to implementation processes, change responses in healthcare, and subcultural resistance to change. Lines: 444, 480, 499. |
| Discussion | Lines 404–406: COVID→change fatigue is interpretation not established finding - rephrase as plausible contributing factor | R8 | Revised Lines 466-470 |
| Discussion | Lines 440–441: 'Adoption depended on…' implies causality - rephrase to 'adoption appeared to be influenced by...' | R8 | Revised Line 525 |
| Discussion | Line 473: prescriptive recommendation from two-site study - rephrase to 'these findings suggest that future implementations may benefit from...' | R8 | Revised Line 585 |
| Discussion | Briefly elaborate which contextual features are specific to Swedish healthcare vs transferable internationally | R7 | Revised Lines 535-540 |

**Limitations**

| **Section** | **Comment** | **Reviewer(s)** | **Action taken** |
| --- | --- | --- | --- |
| Limitations | No audio recordings - discuss threat to credibility and quotation integrity (selective capture, reconstruction bias) explicitly | R1, R3, R6, R7 | Revised Lines 552-559 |
| Limitations | Interviewer positionality - discuss social desirability and analytic bias; acknowledge ED1 vs ED2 differences may reflect interviewer-site effects | R1, R8 | Added a specific subsection. Lines 562-578 |
| Limitations | Cross-site confounding - implementation timing, maturity, COVID-19, concurrent reforms as plausible alternative explanations | R1, R6 | Discussed and revised under subsection contextual explanation for site difference. Line 450-451 |
| Limitations | Cognitive bias as a limitation affecting self-reported preparedness | R1 | Revised Line 493 |
| Limitations | Participant anonymity: Sahlgrenska named + small groups (5 male nurses/site) - confirm anonymity steps; check whether any quotes could be identifying | R8 | We thank the reviewer for raising this important point regarding participant anonymity. Several measures were taken to protect confidentiality beyond the removal of names. No individual quote can be attributed to a specific participant by colleagues, managers, or external readers. The two EDs are large clinical environments, and gender-neutral language is used throughout, meaning individual nurses cannot be distinguished in the dataset. All quotes were carefully reviewed to ensure that no identifying information was retained. |

**Figures / Format**

| **Section** | **Comment** | **Reviewer(s)** | **Action taken** |
| --- | --- | --- | --- |
| Figures | Re-upload all figures with higher resolution | R6 | All figures are revised for readability and to meet journal requirement |
| Format | References at end of sentence throughout manuscript | R5 | Revised throughout the manuscript |
| Format | Reference 39 appears incomplete (line 603); add DOIs where available; ensure all references follow required style | R8 | Reference added correctly |

**Administrative / Other**

| **Section** | **Comment** | **Reviewer(s)** | **Action taken** |
| --- | --- | --- | --- |
| Admin | Reviewer 2: positive review - suggests broadening study to a wider audience | R2 | Thank you. |
| Admin | Data availability statement does not meet journal requirements - specify legal/ethical basis, data controller, formal access process, possibility of de-identified data sharing | R7, R8 | Data availability statement is revised to meet journal requirement. |
| Admin | Ethics approval number discrepancy: line 177 vs Declarations section (line 482) - clarify whether same or separate approvals | R8 | Ethics approval numbers have been revised for consistency. |
