# Supplementary material for: Evaluation of the implementation process of a new emergency triage system: West coast system for triage (WEST)
Source: PLoS One. 2026 Jun 11;21(6):e0350323. doi: 10.1371/journal.pone.0350323 (PMC13258005; doi:10.1371/journal.pone.0350323)
Supplement: Attachment — Submitted filename: Peer Review Report -PLOS.docx [file pone.0350323.s007.docx]

**Peer Review Report**

***Manuscript: PONE-D-26-01867R1***

**Title:** Evaluation of the implementation process of a new emergency triage system: West coast system for triage (WEST)

**Comments to the Editor**

Thank you for the opportunity to review this manuscript. The authors have thoughtfully made meaningful improvements based on the previous reviewer report particularly to the reflexivity statement, the integration of the Diffusion of Innovations framework, and the framing of the cross-site comparison. The study is very impressive and addresses a topic of clear practical relevance.

In my view, the manuscript is acceptable but would benefit from a further round of minor to moderate revision.

**Recommendation: Comments to the Authors**

Thank you for a careful and constructive revision. Your responses to prior reviewers are organized clearly and the manuscript reads more coherently than the earlier version. The following suggestions are offered to further strengthen what is already a useful and timely study.

**Comments:**

**1. Methodological transparency around data collection**

**Line 177-179: The** revised Methods describe "near-verbatim style, however, by definition, real-time written notes during a 30 to 45 minute interview cannot be near-verbatim. The term risks overstating , hence suggestion is to replace near verbatim with detailed contemporaneous notes".

**Line 180:** Please clarify the timing of member checking. Were notes returned to participants immediately after the interview, later the same day, or at a subsequent time? It would also be helpful to indicate the types of revisions typically made.

**Line 695-697:** In the Limitations section, the text moves quickly from noting the absence of recordings to describing mitigating steps. Consider elaborating more directly on what may have been lost without recordings (e.g., hesitation, ambivalence, contradiction, tone) before presenting these mitigations.

**2. Results: presentation and language**

- The phrase "most participants" is used inconsistently. In some places it refers to roughly 35%, in others to over 80%. Use more precise language such as "a majority," "approximately one-third," or "around three-quarters."
- The "Adoption-related attitude patterns" subsection is brief and sits awkwardly between Results and Discussion. Consider either expanding it with concrete illustrative examples of early-adopter and later-adopter response patterns, or moving the material into the Discussion where the interpretive framing is more appropriate.

**3. Naming and abbreviations**

- The track-changes file shows the title was revised from "WEst coast System for Triage" to "West coast system for triage," but the abbreviations list still uses "WEst coast System for Triage" (line 602 of the clean copy). Pick one capitalization convention and apply it everywhere.
- The abbreviations list in the track-changes version includes "ESS: Emergency Symptoms and Signs," but ESS is not used in the clean copy text. Remove if no longer relevant.

**4. References**

- Reference style is inconsistent: some entries include DOI, PMID, and Epub date, others do not.

**5. Few Minor comments**

- Line 211: "data waere coded" appears to be a typo carried over from the track-changes version. Should read "data were coded."
- Abstract Methods: "qualitative descriptive design with a mixed-method approach" should read "mixed-methods" for consistency with the main text.
- Figure 1: the decimal comma in "2,5%" and "13,5%" should be replaced with a decimal point .
- Lines 401–402: "Several participants noted that the implementation coincided with the COVID-19 pandemic" — this applies only to ED2, please clarify.
- AI Disclosure: consider being more specific about which sections were AI-edited, and add an explicit statement that all AI-assisted text was reviewed and verified by the authors.
- Authors' contributions: the clean copy lists "SH, EC, CM, and PAS analyzed data," while an earlier version listed only "SH, EC, and CM." Please confirm which is correct.
- Throughout: "emergency department" is used in lower case in some places and capitalized in others outside the abbreviation. Standardize.

**Summary**

This is a study with practical relevance and a real contribution to make to the implementation literature in emergency care. The authors have responded constructively to a demanding set of prior reviewer comments. With a final round of revision focused on transparency around data collection, the framing of the cross-site comparison, and tightening of the theoretical and presentational elements, the manuscript should be well placed for acceptance**.**

I thank the authors for the careful work they have put into the revision and the editor for the opportunity to review.
