# Supplementary material for: Evaluation of the implementation process of a new emergency triage system: West coast system for triage (WEST)
Source: PLoS One. 2026 Jun 11;21(6):e0350323. doi: 10.1371/journal.pone.0350323 (PMC13258005; doi:10.1371/journal.pone.0350323)
Supplement: Peer review history

# Peer review: Decision Letter 0

**DOI:** 10.1371/journal.pone.0350323.r001

**Authors:** Suzanne Rose

4 Mar 2026

PONE-D-26-01867

Evaluation of the implementation process of a new emergency triage system: WEst coast System for Triage (WEST)

PLOS One

Dear Dr. Habbouche,

Thank you for submitting your manuscript to PLOS ONE. After careful consideration, we feel that it has merit but does not fully meet PLOS ONE’s publication criteria as it currently stands. Therefore, we invite you to submit a revised version of the manuscript that addresses the points raised during the review process.

Please submit your revised manuscript by Apr 18 2026 11:59PM. If you will need more time than this to complete your revisions, please reply to this message or contact the journal office at plosone@plos.org. When you're ready to submit your revision, log on to https://www.editorialmanager.com/pone/ and select the 'Submissions Needing Revision' folder to locate your manuscript file.

Please include the following items when submitting your revised manuscript:

- A letter that responds to each point raised by the academic editor and reviewer(s). You should upload this letter as a separate file labeled 'Response to Reviewers'.
- A marked-up copy of your manuscript that highlights changes made to the original version. You should upload this as a separate file labeled 'Revised Manuscript with Track Changes'.
- An unmarked version of your revised paper without tracked changes. You should upload this as a separate file labeled 'Manuscript'.

-->If you would like to make changes to your financial disclosure, please include your updated statement in your cover letter. Guidelines for resubmitting your figure files are available below the reviewer comments at the end of this letter.

If applicable, we recommend that you deposit your laboratory protocols in protocols.io to enhance the reproducibility of your results. Protocols.io assigns your protocol its own identifier (DOI) so that it can be cited independently in the future. For instructions see: https://journals.plos.org/plosone/s/submission-guidelines#loc-laboratory-protocols. Additionally, PLOS ONE offers an option for publishing peer-reviewed Lab Protocol articles, which describe protocols hosted on protocols.io. Read more information on sharing protocols at https://plos.org/protocols?utm_medium=editorial-email&utm_source=authorletters&utm_campaign=protocols.

We look forward to receiving your revised manuscript.

Kind regards,

Suzanne Rose, MS, PhD

Academic Editor

PLOS One

Journal Requirements:

When submitting your revision, we need you to address these additional requirements.

1. Please ensure that your manuscript meets PLOS ONE's style requirements, including those for file naming. The PLOS ONE style templates can be found at

https://journals.plos.org/plosone/s/file?id=wjVg/PLOSOne_formatting_sample_main_body.pdf and

https://journals.plos.org/plosone/s/file?id=ba62/PLOSOne_formatting_sample_title_authors_affiliations.pdf

2. The ethical approval number(s) listed in the manuscript and/or submission metadata does not match the approval number on the ethical approval document you provided. Please ensure that all approval numbers are correct.

3. Thank you for stating the following financial disclosure:

“Sahlgrenska University Hospital funded the study through the Emergency Development Center and the Gothenburg Emergency Medicine Research Group.”

Please state what role the funders took in the study.  If the funders had no role, please state: "The funders had no role in study design, data collection and analysis, decision to publish, or preparation of the manuscript." If this statement is not correct you must amend it as needed.

Please include this amended Role of Funder statement in your cover letter; we will change the online submission form on your behalf.

4. Please note that funding information should not appear in the Acknowledgments section or other areas of your manuscript. We will only publish funding information present in the Funding Statement section of the online submission form. Please remove any funding-related text from the manuscript.

5. For studies involving third-party data, we encourage authors to share any data specific to their analyses that they can legally distribute. PLOS recognizes, however, that authors may be using third-party data they do not have the rights to share. When third-party data cannot be publicly shared, authors must provide all information necessary for interested researchers to apply to gain access to the data. (https://journals.plos.org/plosone/s/data-availability#loc-acceptable-data-access-restrictions)

For any third-party data that the authors cannot legally distribute, they should include the following information in their Data Availability Statement upon submission:

1) A description of the data set and the third-party source

2) If applicable, verification of permission to use the data set

3) Confirmation of whether the authors received any special privileges in accessing the data that other researchers would not have

4) All necessary contact information others would need to apply to gain access to the data

6. Your ethics statement should only appear in the Methods section of your manuscript. If your ethics statement is written in any section besides the Methods, please delete it from any other section.

7. We note you have included a table to which you do not refer in the text of your manuscript. Please ensure that you refer to Table 1 in your text; if accepted, production will need this reference to link the reader to the Table.

8. If the reviewer comments include a recommendation to cite specific previously published works, please review and evaluate these publications to determine whether they are relevant and should be cited. There is no requirement to cite these works unless the editor has indicated otherwise.

9. Please review your reference list to ensure that it is complete and correct. If you have cited papers that have been retracted, please include the rationale for doing so in the manuscript text, or remove these references and replace them with relevant current references. Any changes to the reference list should be mentioned in the rebuttal letter that accompanies your revised manuscript. If you need to cite a retracted article, indicate the article’s retracted status in the References list and also include a citation and full reference for the retraction notice.

**Additional Editor Comments:**

Thank you for submitting the manuscript to PLOS ONE for review. The article is well written and several excellent comments are available from the reviewers to help guide the revision and resubmission. I had an astoundingly robust response for reviews of this manuscript and therefore, you will see eight different reviewer comments, some of which overlap and can be addressed jointly. I greatly look forward to receiving the revised manuscript.

[Note: HTML markup is below. Please do not edit.]

Reviewers' comments:

Reviewer's Responses to Questions

**Comments to the Author**

1. Is the manuscript technically sound, and do the data support the conclusions?

The manuscript must describe a technically sound piece of scientific research with data that supports the conclusions. Experiments must have been conducted rigorously, with appropriate controls, replication, and sample sizes. The conclusions must be drawn appropriately based on the data presented.

Reviewer #1: Partly

Reviewer #2: Yes

Reviewer #3: Yes

Reviewer #4: Yes

Reviewer #5: Yes

Reviewer #6: Partly

Reviewer #7: Yes

Reviewer #8: Yes

**********

2. Has the statistical analysis been performed appropriately and rigorously?

Reviewer #1: N/A

Reviewer #2: Yes

Reviewer #3: N/A

Reviewer #4: Yes

Reviewer #5: Yes

Reviewer #6: Yes

Reviewer #7: Yes

Reviewer #8: No

**********

3. Have the authors made all data underlying the findings in their manuscript fully available?

The PLOS Data policy requires authors to make all data underlying the findings described in their manuscript fully available without restriction, with rare exception (please refer to the Data Availability Statement in the manuscript PDF file). The data should be provided as part of the manuscript or its supporting information, or deposited to a public repository. For example, in addition to summary statistics, the data points behind means, medians and variance measures should be available. If there are restrictions on publicly sharing data—e.g. participant privacy or use of data from a third party—those must be specified.

Reviewer #1: No

Reviewer #2: No

Reviewer #3: Yes

Reviewer #4: Yes

Reviewer #5: Yes

Reviewer #6: Yes

Reviewer #7: No

Reviewer #8: No

**********

4. Is the manuscript presented in an intelligible fashion and written in standard English?

PLOS ONE does not copyedit accepted manuscripts, so the language in submitted articles must be clear, correct, and unambiguous. Any typographical or grammatical errors should be corrected at revision, so please note any specific errors here.

Reviewer #1: Yes

Reviewer #2: Yes

Reviewer #3: Yes

Reviewer #4: Yes

Reviewer #5: No

Reviewer #6: Yes

Reviewer #7: Yes

Reviewer #8: Yes

**********

5. Review Comments to the Author

Please use the space provided to explain your answers to the questions above. You may also include additional comments for the author, including concerns about dual publication, research ethics, or publication ethics. (Please upload your review as an attachment if it exceeds 20,000 characters)

Reviewer #1: Thank you for the opportunity to review this manuscript. The topic—staff experiences and contextual factors during implementation of a new triage system—has practical relevance for emergency care operations and implementation science. The cross-site design is a strength in principle; however, the manuscript currently overreaches in several interpretations and requires substantially improved methodological transparency to support the conclusions.

Below I outline major and minor issues that should be addressed to improve credibility, clarity, and alignment between data and claims.

Major comments

1) Timeliness / novelty given the age of the dataset

The interviews were conducted April 16–30, 2021, which is now nearly five years ago. Please justify why these findings remain novel and relevant to current practice (e.g., ongoing implementation of WEST, stable implementation barriers), or present the work more explicitly as an implementation case study with enduring lessons rather than contemporary evidence.

Suggested action: Add a short paragraph in the Discussion on the continued relevance of the findings despite the time gap, and clearly delimit which claims are intended to be timeless (e.g., training, leadership engagement, readiness) versus time-sensitive.

2) No audio recordings: major threat to credibility and quotation integrity

A central methodological limitation is the decision not to audio-record interviews. The manuscript indicates that responses were written down “in detail,” later “cleaned” (e.g., removing pauses/fillers), and returned to participants.

This approach introduces substantial risk of:

selective capture during note-taking,

reconstruction bias during “cleaning,” and

overconfidence in quotations that may appear verbatim but are reconstructed.

Suggested action (required):

Use precise terminology (e.g., “detailed interview notes” rather than “transcripts” if not verbatim).

Describe how notes were taken (real-time vs post hoc expansion; whether near-verbatim phrasing was captured; who took notes).

Clarify what “cleaning” entailed and how it could have affected meaning.

Temper the evidentiary weight of quotations accordingly and add a stronger limitations paragraph.

3) Interviewer positionality: risk of social desirability bias and analytic bias

The interviewer was employed as a resident physician at one of the participating EDs. Even without formal managerial authority, this positionality can affect participant openness and the content/tone of responses. It also raises the possibility of differential rapport across sites and potential bias in interpretation.

Required action:

Add a dedicated reflexivity subsection: relationship to participants, potential power dynamics, and mitigation steps (e.g., standardized interview conduct; independent coding/analysis; negative case analysis; external reviewer involvement).

Explicitly acknowledge that differences between ED1 and ED2 may partially reflect interviewer-site effects.

4) Cross-site comparison is heavily confounded (implementation timing, maturity, COVID, concurrent reforms)

ED1 and ED2 differ substantially in timing of implementation, time-since-implementation at the time of interviews, and pandemic context. This makes site comparisons vulnerable to confounding and undermines interpretations framed as differences in “adopter” characteristics.

Required action:

Reframe ED1 vs ED2 differences as context-dependent and exploratory, not as strong evidence of adopter-category differences.

Strengthen the Discussion by explicitly treating “implementation maturity” (time since go-live) and COVID-related instability as plausible primary drivers.

5) Table 1: professional experience differences are plausible explanatory variables but are not integrated

Table 1 suggests differences in years in profession across sites. This could plausibly explain perceptions of preparedness, ease-of-use, clarity, and reliance on clinical judgment—yet the manuscript does not use this information in interpretation.

Required action:

Incorporate staff experience into the analytic narrative as a candidate explanation for site differences.

At minimum, add a sensitivity-style interpretation: “differences may reflect experience mix rather than adopter orientation.”

6) “Accuracy” claims are not supported by objective evidence (e.g., Lines ~190 and ~300)

You report that most participants rated WEST as more accurate than RETTS© in identifying severely ill patients. However, “accuracy” is an outcome term that typically implies comparison against a reference standard (clinical outcomes, deterioration, ICU admission, etc.). In this manuscript, the evidence appears to be perception-based.

Suggested action:

Reword throughout to “perceived accuracy” unless objective performance measures are presented.

Clarify how participants operationalized “accuracy” (e.g., subjective clinical fit, workflow effects, fewer orange categorizations).

Ensure the Discussion does not imply validated superiority without outcomes data.

7) ED1 higher Likert ratings than ED2: consider staff mix and novice/seniority as alternative explanations (Line ~195)

You note that ED1 respondents tended to report higher Likert ratings than ED2 respondents. Please consider that site differences might reflect differences in staff composition (age/experience), novelty effects, or local implementation maturity rather than adopter category per se.

Suggested action: Explicitly discuss whether ED1 respondents represented a younger/more novice cohort and how this might influence perceptions (including possible optimism bias or different expectations).

8) Training, feedback opportunities, and preparedness: large site differences need deeper explanation (Lines ~232 and ~252)

You report substantial differences between sites regarding time for training, feedback opportunities, and preparedness (e.g., 76% vs 32%; 82% vs 32%). The manuscript currently reports these differences but does not adequately investigate why they exist.

Required action:

Provide contextual detail: Were training modalities genuinely different? Was staffing/coverage different? Was management engagement different?

Avoid interpreting this primarily as “late adopters” vs “early adopters” unless you have direct evidence.

9) Management involvement and organizational readiness: consider more suitable theory or stronger justification (Lines ~228, ~398, ~432)

Several quotations and themes appear to align more strongly with organizational readiness, leadership engagement, implementation climate, and change management rather than diffusion attributes alone. Additionally, at least one section juxtaposes manager reports (“training was organized similarly”) with nurse perceptions (“superficial”), without clear triangulation.

Required action:

Explain how discrepancies between manager and staff accounts were handled analytically.

Either strengthen your mapping to Rogers (with clear logic and evidence) or justify/augment with a complementary implementation framework (e.g., CFIR, NPT, or organizational readiness theory).

If you retain Rogers, explicitly show how your themes align with its constructs and what your data suggests about sequencing (i.e., how X precedes Y), rather than asserting adopter categories.

10) Clinical judgment demands, experience, and possible cognitive bias (Lines 281, 347, 357)

You report that WEST places higher demands on nurses with less clinical experience. Please clarify whether experience level was linked to respondents in a way that supports this conclusion analytically, or whether it is a perception expressed by participants.

In addition, perceived preparedness or competence can be affected by cognitive bias (e.g., overconfidence among novices, limited feedback loops).

Suggested action:

Present this primarily as a perceived risk unless you have analytic linkage to experience.

Consider acknowledging cognitive bias as a limitation affecting self-reported preparedness.

11) Incomplete Likert reporting (Line 287 and similar instances)

Some results report only a subset of response categories (e.g., “clearer” and “equally clear”) without stating what the remaining respondents selected.

Suggested action (required):

Report complete distributions for each Likert item (either in text, a table, or supplementary material).

If you collapse categories, justify the approach and provide the full distribution elsewhere.

12) “Over-triage” language is potentially misleading (Line ~325)

The manuscript implies reduced “over-triage” based on fewer orange categorizations. Without objective outcomes or a reference standard, a reduction in orange assignments is not necessarily true “over-triage reduction”; it may reflect different calibration/thresholds between systems.

Suggested action (required):

Replace “over-triage” with more precise process language (e.g., “reduced assignment to orange” or “shift in triage category distribution”), unless validated outcomes are presented.

If you have prior pilot data, ensure you clearly distinguish what is measured externally versus perceived here.

13) Practical implication: experience-dependent triage may be a real operational risk (Lines 347 and 357)

If effective use of WEST requires substantial clinical experience, this may be a significant operational issue in EDs with high turnover or less experienced staff. The Discussion should address whether the system is appropriate across staffing contexts and what safeguards (training, supervision, decision support) are needed.

Minor comments

Ethics identifiers: Please ensure ethical approval identifiers are consistent throughout the manuscript.

Data availability statement: For qualitative interview notes, clarify restrictions in terms of confidentiality/re-identification risk and provide a clear access pathway (e.g., data access committee), rather than broad “licensing” phrasing unless applicable.

Mixed-methods clarity: The Likert component reads as add-on/descriptive. Either integrate it more formally (e.g., joint displays, explicit mixing rationale) or clearly position it as supportive descriptive context.

Coding transparency: Provide clearer detail on coding procedures (portion double-coded, codebook development, handling disagreements, negative cases).

Concluding recommendation to the authors

I understand that reading this review and seeing the number of suggested changes might feel discouraging. Please know that I took the time to read your work carefully, and my feedback is intended to support you and help strengthen the study.

However, the manuscript will require substantial revision. In particular, greater methodological transparency is needed given the absence of recordings; the suitability of the chosen theoretical framework should be reconsidered or supplemented with additional theories to strengthen the analysis and conclusions; and the sections on reflexivity/positionality, potential confounding in cross-site comparisons, and the framing of “accuracy” and “over-triage” as perceptions all need further development. With these revisions, the manuscript has the potential to offer valuable implementation insights.

As it stands, however, the strength of the conclusions exceeds what the data can support from my perspective.

I wish you the best with your revisions and hope you find this feedback helpful.

Reviewer #2: I would like to congratulate the authors for the study performed and also for the clarity and comprehensiveness of the presentation of the results, analyses, limitations and conclusions. Would be beneficial to broaden the study among greater audience.

Reviewer #3: Overall

The manuscript presents a clear rationale for revising triage practices in light of ED crowding, a well-described two-site implementation context with transparent qualitative methods, and coherent results that link cross-site differences to concrete implementation domains. These findings are brought together in a discussion that draws effectively on foundational theory to interpret heterogeneous uptake and generate practical recommendations.

Success of clinical decision support tools such as triage systems relies on moving from abstract theory to routine clinical practice. Successful implementation and sustained adoption are shaped by staff perceptions of value, trust in the tool’s reliability, and usability within real-world workflows. A particular strength of this study is its focus on frontline staff perspectives, which provides actionable insights to strengthen future implementations.

The feedback below is offered to further strengthen this important work by clarifying key claims, reinforcing the links between theory and findings, and sharpening the implementation lessons to be as clear and actionable as possible.

Background

1. Overall, you do a nice job of outlining the problem statement and relevance and tying that to your aims

2. You move from concerns about RETTS assignment of acuity to over-triage in the next sentence. It would help to add a linkage between these two concepts – how does the acuity relate to the over-triage?

3. Also there is an abrupt change between RETTS/WEST and organizational culture. Would recommend adding a transitional sentence or two – something like “While the rationale for developing WEST was grounded in concerns about over-triage and consistency in RETTS, introducing a new triage system requires substantial changes to workflows, roles, and shared clinical judgment. Accordingly, the success of WEST depends not only on its design but also on how it is implemented and adopted in everyday ED practice.”

4. Line 88 and 89 the wording is a little confusing – would revise to something like “Rogers’ concepts are particularly useful in EDs because EDs function as unique social….”

5. At lines 96/97 you transition from Rogers to Moore, however this is not clear in the text if you are not familiar with these works. Would recommend:

a. End this sentence after the references. Furthermore, Rogers’ “Diffusion of Innovations” offer concepts for understanding adoption within a social system (28, 33, 34).

b. Add a sentence that discusses how moving from initial uptake into broad routine use is often difficult, particularly at the transition from early adopters to the early majority and note that Moore terms this challenge “Crossing the Chasm”

c. Here is an example – “A recurring implementation challenge is the shift from early adopters to the early majority, which Moore describes as “Crossing the Chasm”.

6. It would be nice to conclude the framework section by tying it back to your study. You start by Saying that “Diffusion of Innovations” was a framework. How was the “Crossing the Chasm” incorporated into your design? Did you evaluate perceptions from early adopters separately? If it wasn’t used in your design then I would recommend moving this to the discussion.

7. Your aims are good, but consider whether you’ll truly be able to speak to “accuracy” from nurse perceptions alone. Would recommend saying “perceived accuracy” or speak to “clinical appropriateness” or “nurse confidence” instead.

8. A few minor grammatical recommendations:

• “demand often exceeded capacity” change to “demand often exceeds capacity”

• “Improvement efforts therefore targeted ED flow” change to “Improvement efforts have therefore targeted ED flow”

• “reduction on over-triage” change to “reduction in over-triage”

• Remove the extra comma between “ED context” and references (17, 26)

• “introducing new triage system” change to “introducing a new triage system”

• “In a literature review of process improvements approaches…” change to “process improvement approaches”

• “offer concepts” change to “offers concepts”

Methods

1. You do a nice job describing the setting and eligibility criteria. Dual coding strengthens credibility of the study and your cross-case logic is well explained.

2. You write the HTA “aimed to identify triage systems effective in preventing ED crowding.” That’s a strong causal claim for triage alone. Consider softening to something like “effective in supporting patient prioritization and ED flow under crowded conditions”

3. You state “ED2 had longer length-of-stay and time-to-physician”, however provide no source for where this was established. Was this observed in the HTA? If so, make this clear and move it to the paragraph discussing the HTA. If not, provide source and context.

4. On line 123 when you state “in this study” are you referring to the HTA study or the present study?

5. Would recommend reframing the paragraph starting at line 125.

a. Something like “Based on the HTA findings, WEST was selected as a promising approach to support patient flow at ED1 and ED2 under crowded conditions by combining a broader assessment framework with explicit room for clinical judgment. WEST was implemented….”

b. I would remove the stroke scale and coma scale as it doesn’t seem to be relevant.

6. This verbiage is also a little unclear “At the time of the study, WEST was available in a pocket-sized version” – would recommend something like “pocket-sized WEST reference card” that makes it clear what is available.

7. Provide more detail for the transcription – who transcribed, how was it transcribed –typed into a form, handwritten, how were quotes handled?

8. Were there any missing responses? If so, how were they handled?

9. At line 158 you mention unexpected themes emerging from the last question – expand on how you identified and grouped these themes

10. A few minor grammatical recommendations:

• “one of Europe’s largest and Sweden’s largest hospital” consider changing to “one of Europe’s largest hospitals and the largest in Sweden”

• “cleaned of pauses, overlaps, and fillers” mainly apply to audio transcripts; with notes, it sounds odd. Consider something like “edited for readability while preserving wording.”

Results

1. Incorporating direct quotes/perceptions in addition to the Likert scale results is powerful and provides great insight for others who may be interested in rolling out a new triage system.

2. Add a response rate. It appears to be 36 of 72 eligible triage nurses participated (50%).

3. Line 190 refers to Question 7, which does not provide a lot of information to a reader who doesn’t have the questionnaire in front of them. Perhaps reword as “Responses from ED2 tended to be more variable across participants, however, when evaluating perceived accuracy most participants across both sites rated WEST as more accurate than RETTS© in identifying severely ill patients.”

4. Likert results should be more consistent in wording and in alignment with the likert scale. Either label as levels 4 or 5 or agree/strongly agree. Examples include:

• “Most participants… considered information sufficient (76%)” – is sufficient a 4 or 5? If so, change to something like “Most participants (76%) agreed or strongly agreed that the education provided on the new triage system was sufficient.”

• “30 considered WEST more accurate” change to something like “Of the 36 participants, 30 agreed or strongly agreed that WEST was more accurate than RETTS in identifying severely ill patients…..”

5. Adoption patterns section feels thin given the emphasis in the background. Recommend adding a dedicated Adoption Patterns section.

a. who sounded like early adopters/what did early adopter sound like (e.g., expressed enthusiasm, experimentation, advocacy)?

b. who sounded like early majority/what did early majority sound like (wanted evidence, clearer guidelines)

c. How specifically did these vary by site?

6. Minor grammatical recommendations

• “Participants from ED2 were of higher age” use “were older” instead

• “reported a longer period of employment” use “reported longer tenure” instead

• “Gender distribution was similar (ED1 12/5; ED2 14/5 female/male).” Perhaps replace with “Gender distribution (female/male) was similar between ED1 (12/5) and ED2 (14/5).” for clarity.

• “Participants’ perceptions of that education” change to “of the education”

• “Most participants perceived WEST as both more flexible and more accurate” replace with “perceived WEST to be…”

7. It would be helpful to add a summary of cross-site differences at the end of each section. For example, after Implementation something like “Across domains, ED1 reported more structured communication, interactive training, and preparedness.”

Discussion

1. Overall, well done. Content is appropriate and includes clear summary of results, interpretation of adoption in the context of Rogers/Moore frameworks, and actionable implications.

2. Consider if there is an opportunity to further link your findings to the specific attributes from the Rogers work that you measured:

a. Relative advantage: reduced orange over-prioritization / fewer labs (perceived)

b. Complexity: harder for novices; lack of comprehensive handbook; no digital tool

c. Compatibility: blood sampling routines; integration with other units/physicians

d. Communication channels: rumors vs structured briefings

3. Add reference for expansion of WEST at line 446 “In the case of WEST, the innovation not only overcame an early setback but has since expanded across multiple healthcare regions.” Also, what is the early setback that is being referred to?

4. Line 449 make it clear that this study underscores the risks new innovations face during the initial phases of dissemination

5. You say essentially the same “training designed similarly but perceived differently” point twice (lines ~399–403 and ~421–425). Keep it once, and use the other spot to add something new (e.g., presence of champions).

6. In the Limitations be more explicit on the limitations of using written notes rather than audio recordings (e.g., exactness of quotations, ability to capture tone and emphasis)

7. At line 392 “demonstrated faster and more positive adoption” implies you measured adoption speed, recommend changing to “showing more rapid and positive adoption” to soften the language

8. You mentioned the interviewer in ED2 was a resident. Did the participants in ED1 know the interviewer? Is this a potential source of bias in responses (e.g., did ED2 participants share more freely)?

Reviewer #4: The manuscript is well prepared, has followed all required research processes and obtained ethical approval, and is acceptable for publication. However, there are minor corrections that need to be addressed by the researchers as outlined in the attached file.

Reviewer #5: Thank you for the opportunity to review this important paper. The issues facing the implementation of new triage systems, especially in the dynamic environment of an ED, are complex and multifactorial. This paper has the potential of offering important insights into concrete staff experiences. Further, the qualitative methodology is a useful but under-utilized tool in Emergency Medicine. Overall, the methodology, results and conclusions of the paper are sound. However, there are a number of issues, principally relating to the language and layout of the paper, that need to be worked on before it can be considered ready for publication.

The text is at times unnecessarily convoluted and cumbersome to read. This distracts from the content and makes the manuscript less accessible. Examples include the following sentence “Rogers' concepts of early and late adopters in healthcare change have been applied in studies on social system levels on a large scale (29), in selected diagnosis groups (30), as well as in smaller units and populations within healthcare (31). Some reasons to use Rogers' concepts on EDs exclusive include the fact that EDs are unique social systems and operates within its own specific contextual factors, including staff dynamics, leadership styles, patient demographics, and resource availability. Further, the sentence “Participants’ perceptions of that education differed substantially between sites.” Is unintelligible. It is essential that a native English speaker reviews the manuscript for readability and grammar so that the important messages of this study are presented in correct academic English.

Further, I would question the utility of Roger’s Diffusion of Innovations (DoI) in this case. The stated aims of the study are “(i) to explore how triage nurses experienced the transition from RETTS© to WEST, and (ii) to describe perceived benefits and drawbacks of WEST relative to RETTS© regarding flexibility, clarity, and accuracy. We also compared implementation processes between ED1 and ED2.” No mention is made of identifying late adopters etc, which is then mentioned in the Discussion (but not results). While this is not make-or-break for acceptance, I think removing the parts about DoI makes for a leaner and more succinct paper.

A few additional points below:

Line 49: What is a “frontline nurse”?

Line 61: Spell out NEWS2

Lines 67-70 (and rest of manuscript): Put references at end of sentence throughout.

Please state study design and type of study as a first sentence in the methods.

Line 149: avoid personal pronouns

Consider filling out the COREQ guidelines for qualitative studies and include as an appendix.

Table 1: Interquartile range, not “range”.

162-163: Likert-scale responses were summarized using proportions of high ratings, defined as scores of 4-5 on a five-point scale: please put the %s in the figure in addition to mentioning them in the text.

Again, a very good paper, appropriate for publication pending the above revisions. Looking forward to reading a new draft.

Reviewer #6: DearAuthor

This manuscript evaluates the implementation of a new emergency triage system (WEST) in two Swedish emergency departments using Rogers' "Diffusion of Innovations" as a theoretical framework. The study is methodologically strong with a clear comparative design, appropriate mixed-methods approach, and rich qualitative data from 36 nurses. The findings that implementation success depends on context, communication, education, and organizational readiness are well-supported and practically valuable.

The methodological limitations require more prominent discussion, including the absence of audio recording during interviews, the interviewer's dual role as a clinician at one study site, and the substantial difference in implementation-to-interview timing between sites. According to the manuscript , ED1 was interviewed 17 months post-implementation while ED2 was interviewed only 6 months post-implementation, which may have influenced responses and limits direct comparability. Additionally, the quality of figures included at the end of the manuscript is very poor and must be re-uploaded with higher resolution. Despite these concerns, the theoretical framework is well-applied, the qualitative analysis is rigorous, and the practical implications are significant.

Reviewer #7: Dear Authors,

This manuscript presents a well-conducted qualitative study exploring the implementation of the WEST triage system in two emergency departments. The topic is relevant to emergency medicine and implementation science, and the use of a theoretical framework (Diffusion of Innovations) provides a structured lens for interpretation. The study design aligns with the stated aims, and the conclusions are cautious and generally supported by the data presented.

The qualitative methodology is appropriate for examining implementation processes. The sample size (n=36) is reasonable for this type of inquiry, and the use of independent coding and member checking strengthens analytic credibility. The integration of descriptive Likert-scale data with qualitative findings is consistent with the exploratory design.

However, several issues should be addressed to strengthen methodological transparency and compliance with journal policy:

1. Qualitative Data Collection and Rigor

Interviews were not audio-recorded; instead, detailed written notes were taken and returned to participants for confirmation. While member checking is a strength, the absence of verbatim transcription may limit analytic depth and transparency. Please clarify:

• How completeness and accuracy of note-taking were ensured;

• How potential loss of nuance was mitigated;

• Whether any structured approach was used to standardize documentation.

This limitation should also be discussed explicitly in relation to qualitative rigor.

In addition, the manuscript would benefit from explicit reference to established qualitative reporting standards (e.g., COREQ), including a clearer discussion of reflexivity and the interviewer’s positionality.

2. Statistical Component

The quantitative component consists of descriptive summaries of Likert-scale responses. This approach is appropriate given the exploratory and primarily qualitative design. However, the Methods section should explicitly state that no inferential statistical testing was planned or performed due to the study design and sample size, in order to avoid potential misinterpretation.

3. Data Availability

The current Data Availability Statement indicates that supporting data are not publicly available due to licensing restrictions and may be shared upon reasonable request and additional ethics approval.

Under the PLOS Data Policy, “available upon request” is generally insufficient unless a formal and transparent access mechanism is clearly specified. The manuscript should clarify:

• The legal or ethical basis for restricting public access;

• The identity of the data controller;

• The formal process through which qualified researchers may request access;

• Whether de-identified qualitative data could be shared in a repository.

Revision of the Data Availability Statement will be necessary to ensure compliance with journal requirements.

4. Transferability and Context

The discussion appropriately emphasizes contextual differences between ED1 and ED2. It would further strengthen the manuscript to briefly elaborate on which contextual features are likely specific to the Swedish healthcare system and which may be transferable to other emergency care settings internationally.

Overall, this is a thoughtful and methodologically appropriate study addressing an important operational issue in emergency care. With clarification of qualitative rigor and data availability procedures, the manuscript would meet the publication criteria of PLOS ONE.

Reviewer #8: The current manuscript addresses a very important and relevant study addressing implementation of triage innovation in the context of emergency department. The research question is clinically relevant, the two-site comparison is a strength mixed approach is appropriate. Minor revisions and clarifications have to be justifies and is been uploaded

**********

6. PLOS authors have the option to publish the peer review history of their article (what does this mean?). If published, this will include your full peer review and any attached files.

If you choose “no”, your identity will remain anonymous but your review may still be made public.

**Do you want your identity to be public for this peer review?** For information about this choice, including consent withdrawal, please see our Privacy Policy.

Reviewer #1: No

Reviewer #2: **Yes:** Professor Rostislav Kostadinov MD, PhD, DSc

Reviewer #3: No

Reviewer #4: **Yes:** Dr. Fatuma Aden Affey

Reviewer #5: No

Reviewer #6: **Yes:** Hamidreza Aghababaeian

Reviewer #7: **Yes:** NINELL KADYROVA

Reviewer #8: **Yes:** Dr.Manju Avinash Nair

**********

[NOTE: If reviewer comments were submitted as an attachment file, they will be attached to this email and accessible via the submission site. Please log into your account, locate the manuscript record, and check for the action link "View Attachments". If this link does not appear, there are no attachment files.]

To ensure your figures meet our technical requirements, please review our figure guidelines: https://journals.plos.org/plosone/s/figures

You may also use PLOS’s free figure tool, NAAS, to help you prepare publication quality figures: https://journals.plos.org/plosone/s/figures#loc-tools-for-figure-preparation.

NAAS will assess whether your figures meet our technical requirements by comparing each figure against our figure specifications.

Attachment Submitted filename: Evaluation of of WEEST coast System.docx

Attachment Submitted filename: Reviewer suggestions.docx

# Author comment: Author response to Decision Letter 1

**DOI:** 10.1371/journal.pone.0350323.r002

17 Apr 2026

Response to reviewers

Dear Editor,

We would like to express our sincere gratitude to the Academic Editor and the eight reviewers for the opportunity to revise our manuscript. Your comments were thoughtful and constructive and have meaningfully strengthened the work. Although we have not repeated our appreciation in each individual response, we are grateful for every comment received. Also, we have organized our responses thematically rather than addressing each comment individually. We hope this approach is acceptable and remain happy to address any further questions or comments.

Sincerely,

The Authors

Please see attached file "Response to reviewers" for details

Attachment Submitted filename: Response_to_reviewers.docx

# Peer review: Decision Letter 1

**DOI:** 10.1371/journal.pone.0350323.r003

**Authors:** Suzanne Rose

13 May 2026

Evaluation of the implementation process of a new emergency triage system: West coast system for triage (WEST)

PONE-D-26-01867R1

Dear Dr. Habboush,

We’re pleased to inform you that your manuscript has been judged scientifically suitable for publication and will be formally accepted for publication once it meets all outstanding technical requirements.

Within one week, you’ll receive an e-mail detailing the required amendments. When these have been addressed, you’ll receive a formal acceptance letter and your manuscript will be scheduled for publication.

An invoice will be generated when your article is formally accepted. Please note, if your institution has a publishing partnership with PLOS and your article meets the relevant criteria, all or part of your publication costs will be covered. Please make sure your user information is up-to-date by logging into Editorial Manager at Editorial Manager® and clicking the ‘Update My Information' link at the top of the page. For questions related to billing, please contact billing support.

If your institution or institutions have a press office, please notify them about your upcoming paper to help maximize its impact. If they’ll be preparing press materials, please inform our press team as soon as possible -- no later than 48 hours after receiving the formal acceptance. Your manuscript will remain under strict press embargo until 2 pm Eastern Time on the date of publication. For more information, please contact onepress@plos.org.

Kind regards,

Suzanne Rose, MS, PhD

Academic Editor

PLOS One

Additional Editor Comments (optional):

Thank you for your thoughtful revisions - when submitting the final document, please see a few additional modifications requested from the reviewers below.

Reviewers' comments:

Reviewer's Responses to Questions

**Comments to the Author**

1. If the authors have adequately addressed your comments raised in a previous round of review and you feel that this manuscript is now acceptable for publication, you may indicate that here to bypass the “Comments to the Author” section, enter your conflict of interest statement in the “Confidential to Editor” section, and submit your "Accept" recommendation.

Reviewer #1: All comments have been addressed

Reviewer #2: All comments have been addressed

Reviewer #3: All comments have been addressed

Reviewer #5: All comments have been addressed

Reviewer #8: All comments have been addressed

**********

2. Is the manuscript technically sound, and do the data support the conclusions?

The manuscript must describe a technically sound piece of scientific research with data that supports the conclusions. Experiments must have been conducted rigorously, with appropriate controls, replication, and sample sizes. The conclusions must be drawn appropriately based on the data presented.

Reviewer #1: Yes

Reviewer #2: Yes

Reviewer #3: Yes

Reviewer #5: Yes

Reviewer #8: Yes

**********

3. Has the statistical analysis been performed appropriately and rigorously?

Reviewer #1: Yes

Reviewer #2: Yes

Reviewer #3: Yes

Reviewer #5: Yes

Reviewer #8: Yes

**********

4. Have the authors made all data underlying the findings in their manuscript fully available?

The PLOS Data policy requires authors to make all data underlying the findings described in their manuscript fully available without restriction, with rare exception (please refer to the Data Availability Statement in the manuscript PDF file). The data should be provided as part of the manuscript or its supporting information, or deposited to a public repository. For example, in addition to summary statistics, the data points behind means, medians and variance measures should be available. If there are restrictions on publicly sharing data—e.g. participant privacy or use of data from a third party—those must be specified.

Reviewer #1: Yes

Reviewer #2: Yes

Reviewer #3: Yes

Reviewer #5: Yes

Reviewer #8: Yes

**********

5. Is the manuscript presented in an intelligible fashion and written in standard English?

PLOS ONE does not copyedit accepted manuscripts, so the language in submitted articles must be clear, correct, and unambiguous. Any typographical or grammatical errors should be corrected at revision, so please note any specific errors here.

Reviewer #1: Yes

Reviewer #2: Yes

Reviewer #3: Yes

Reviewer #5: Yes

Reviewer #8: Yes

**********

6. Review Comments to the Author

Please use the space provided to explain your answers to the questions above. You may also include additional comments for the author, including concerns about dual publication, research ethics, or publication ethics. (Please upload your review as an attachment if it exceeds 20,000 characters)

Reviewer #1: (No Response)

Reviewer #2: (No Response)

Reviewer #3: I appreciate the authors’ careful attention to the reviewers’ feedback. This is a technically sound descriptive study that focuses on capturing insights from frontline staff for operationalizing the WEST clinical decision-support tool for ED triage. My previous concerns have been addressed in the latest draft. I have outlined a few minor suggestions and grammar edits to further polish the manuscript for publication.

Line 333-334 “Information was also lacking for physicians” sounds like a claim from your results. Would suggest modifying to something like “Participants also reported that information…”

Line 545: Would recommend saying “while ED2 responses were more heterogeneous and included more cautious views resembling those associated with the early or late majority categories.”

Would recommend adding to the “Contextual explanations for site differences” section a statement on potential self-selection bias. The response rate was 46%, with self-selected participation. This introduces a plausible risk that respondents were more engaged, more critical, or more motivated than non-respondents. Since attitudes toward implementation are central to the paper, the manuscript could benefit from a short discussion of how self-selection may have influenced findings. For example, nurses with strong positive or negative views may have been more likely to participate. The authors should also clarify whether participants differed from nonparticipants in seniority if that information is known.

If there is any further information on the education component as a Supplemental document as that information may be helpful to others deploying WEST.

A few nitpicky edits for spelling/grammar as well as suggestions to improve clarity and flow.

Line 52/53: "Rising attendances and suboptimal use of hospital resources means" should be "Rising attendances and suboptimal use of hospital resources mean"

Line 55: "data was collected" should be "data were collected"

Line 76: Would suggest modifying to be "where a single abnormal parameter could escalate the acuity level" to be more precise.

Line 83: "Organizational changes in healthcare often fails" should be "Organizational changes in healthcare often fail."

Line 84: "Subcultures could resist new systems" should be "Subcultures can resist new systems"

Line 97: would recommend rephrasing as "difficulty generalizing results from other areas"

Line 109: "how financial rewards influences" should be "how financial rewards influence"

Line 187: would recommend clarifying as "The educational intervention was designed to be identical at both sites"

Line 306: “On Likert-scale summaries” would recommend to rephrase as “Descriptive summaries of Likert-scale responses showed ED….”

Line 471: “At ED1 71%” should be “At ED1, 71%”

Line 565: would suggest changing “more years in the profession” to “more years of professional experience”

Line 675: “Sweden's tax-funded system” would be more clear to say “Sweden's tax-funded healthcare system” although it is implied.

Reviewer #5: Well done addressing comments from a large amount of reviewers. The manuscript is much clearer. Happy to accept for publication.

Reviewer #8: Minor modifications as per the attachment to be caried out to further strengthen what is already a useful and timely study.

**********

7. PLOS authors have the option to publish the peer review history of their article (what does this mean?). If published, this will include your full peer review and any attached files.

If you choose “no”, your identity will remain anonymous but your review may still be made public.

**Do you want your identity to be public for this peer review?** For information about this choice, including consent withdrawal, please see our Privacy Policy.

Reviewer #1: **Yes:** Daniel Wittrock

Reviewer #2: **Yes:** Rostislav Kostadinov, MD, PhD, DMS

Reviewer #3: No

Reviewer #5: **Yes:** Maximilian P. Nerlander

Reviewer #8: **Yes:** Dr.Manju Avinash Nair

**********

Attachment Submitted filename: Peer Review Report -PLOS.docx

# Editor report: Acceptance letter

**DOI:** 10.1371/journal.pone.0350323.r004

**Authors:** Suzanne Rose

PONE-D-26-01867R1

PLOS One

Dear Dr. Habbouche,

I'm pleased to inform you that your manuscript has been deemed suitable for publication in PLOS One. Congratulations! Your manuscript is now being handed over to our production team.

At this stage, our production department will prepare your paper for publication. This includes ensuring the following:

* All references, tables, and figures are properly cited

* All relevant supporting information is included in the manuscript submission,

* There are no issues that prevent the paper from being properly typeset

You will receive further instructions from the production team, including instructions on how to review your proof when it is ready. Please keep in mind that we are working through a large volume of accepted articles, so please give us a few days to review your paper and let you know the next and final steps.

Lastly, if your institution or institutions have a press office, please let them know about your upcoming paper now to help maximize its impact. If they'll be preparing press materials, please inform our press team within the next 48 hours. Your manuscript will remain under strict press embargo until 2 pm Eastern Time on the date of publication. For more information, please contact onepress@plos.org.

You will receive an invoice from PLOS for your publication fee after your manuscript has reached the completed accept phase. If you receive an email requesting payment before acceptance or for any other service, this may be a phishing scheme. Learn how to identify phishing emails and protect your accounts at https://explore.plos.org/phishing.

If we can help with anything else, please email us at customercare@plos.org.

Thank you for submitting your work to PLOS ONE and supporting open access.

Kind regards,

PLOS ONE Editorial Office Staff

on behalf of

Dr. Suzanne Rose

Academic Editor

PLOS One
